# Work instability, musculoskeletal disorders and absenteeism among Primary Care nurses*

**DOI:** 10.1590/1518-8345.8307.4939

**Published:** 2026-10-07

**Authors:** Heloisa Ehmke Cardoso dos Santos, Rafael de Souza Petersen, Fernanda Ludmilla Rossi Rocha, Lacir José Santin, Maria Helena Palucci Marziale

**Affiliations:** 1 Universidade de São Paulo, Escola de Enfermagem de Ribeirão Preto, PAHO/WHO Collaborating Centre for Nursing Research Development, Ribeirão Preto, SP, Brazil.; 2 Fundação Oswaldo Cruz, Brasília, DF, Brazil.

**Keywords:** Job Security, Cumulative Trauma Disorders, Primary Health Care, Nursing, Occupational Health, Absenteeism

## Abstract

**Objective:**

to analyze the association between work instability, musculoskeletal disorders and absenteeism in primary health care nurses.

**Method:**

a methodological, cross-sectional, quantitative study conducted with 286 primary health care nurses. The Sociodemographic and Occupational Questionnaire and the Brazilian version of the Nurse-Work Instability Scale were used. Data collection was performed via a digital platform and analyzed using descriptive statistics, statistical tests, and regression models (p<0.05). The Strengthening the Reporting of Observational studies in Epidemiology (STROBE) guidelines were used to prepare the study.

**Results:**

286 nurses participated in the study, however, 143 (50.0%) presented work instability, with 9.8% at low risk, 32.9% at medium risk, and 57.3% at high risk. There was a significant association between high instability and musculoskeletal symptoms, including pain, heaviness, and fatigue, affecting multiple body regions. Greater instability was noted among professionals with higher family income. Musculoskeletal disorders were the primary cause of absenteeism, although there was no statistically significant association between instability and leave of absence.

**Conclusion:**

instability at work among primary health care nurses is prevalent and associated with occupational and physical factors, necessitating interventions to preserve work capacity.

## Highlights

**(1)** Study analyzed work instability and musculoskeletal pain in Primary Care.

**(2)** Instability was associated with neck pain and higher absenteeism among nurses.

**(3)** High instability was associated with pain, heaviness and fatigue in various regions of the body.

**(4)** Musculoskeletal symptoms were the main cause of sick leave.

**(5)** Results impact health policies and work management in nursing.

## Introduction

Human labor is one of the main drivers of social and economic development, providing individuals not only with subsistence, but also with important social relationships and opportunities for professional growth. In this context, occupational health and safety, as well as employees’ functional capacity, are decisive factors for the sustainability of productive systems and social well-being^1^.

Nursing, as a central profession in the health field, plays a fundamental role in promoting quality of life and comprehensive care for people, acting at different levels of the health system, especially in Primary Health Care (PHC)^2^.

In Brazil, nursing professionals constitute the second-largest category of higher-education workers in health, and are essential for the implementation of public policies and for ensuring universal and comprehensive access to health services^3^. However, these professionals often face work overload, multiple roles, and increasing demands, factors that increase the risk of physical and mental illness, especially in PHC, where working conditions and organizational challenges directly impact their health^4^. In addition, an international study has shown that factors such as long working hours, increased physical workload, and decreased professional autonomy can increase vulnerability to the onset of musculoskeletal disorders, highlighting the influence of socioeconomic context on their occurrence^5^.

Occupational risks in nursing encompass physical, chemical, and biological factors, as well as ergonomic and psychosocial risks. Of particular note are work-related musculoskeletal disorders (WRMD), caused by poor posture, repetitive movements, and long working hours, which result in functional limitations and impair work capacity^6^. At the same time, psychosocial risks, such as work stress, harassment, insecurity, and lack of support, have been recognized as important factors in mental illness among nurses, contributing to burnout syndrome, anxiety, and depression^7^.

In the work environment, the period preceding work disability may begin when the worker experiences difficulties performing work activities, necessitating intervention at this point to prevent leave and even job loss^8^ ^)-(^ ^9^. Thus, work instability can be seen as a transitory and potentially reversible state if identified and treated early, thereby promoting psychosocial and economic benefits for the worker and the employing institution^9^, minimizing absences, and reducing work capacity^10^.

Given the relevance of nursing in PHC and the adverse effects of working conditions on the health and productivity of these professionals, it is essential to investigate work instability, particularly with respect to musculoskeletal disorders and absenteeism. Although there are studies on occupational risks and musculoskeletal disorders in nurses, work instability is still a little-explored topic, especially in PHC. This knowledge gap limits our understanding of how precarious employment relationships and unstable working conditions can affect the physical and mental health of these professionals, impacting their ability to work and leading to absenteeism.

Thus, this study aimed to examine the associations among work instability, musculoskeletal disorders, and absenteeism among nurses in Primary Health Care.

## Method

### Study design

This study was conducted in two procedural stages: the first stage comprised a methodological study to construct and validate a questionnaire and the second comprised an observational, descriptive, cross-sectional study with a quantitative data analysis. The Strengthening the Reporting of Observational studies in Epidemiology (STROBE) guidelines were used to prepare the study.

The first stage involved the construction and validation of a sociodemographic and occupational questionnaire for primary health care nurses, based on the references of Polit and Beck^11^ and Pasquali^12^. Initially, a preliminary version of the instrument was developed based on a review of literature and theoretical principles of items formulation, structure, and semantics. Then, the instrument was submitted for content validation by a committee of seven experts with experience in occupational health, musculoskeletal disorders, and mental health. Validation was conducted in three rounds, with analysis of item relevance and calculation of the Content Validity Index (CVI), yielding a final CVI of 1.0. Subsequently, a pre-test was conducted with 30 PHC nurses to verify the clarity, applicability, and semantic and cultural equivalence of the sociodemographic and occupational questionnaire. The pre-test participants were included in the final study sample and the results confirmed the questionnaire’s suitability for large-scale use. The second stage consisted of an observational, descriptive and cross-sectional study with a quantitative approach to the data, in which the following questionnaires were used for data collection: sociodemographic and occupational questionnaire (Quest APS - sociodemographic occupational) and Nursing Work Instability Scale (EIT-nursing).

### Study location

Primary Health Care Units in the State of São Paulo, Brazil, from July 27, 2023, to September 30, 2023.

### Population and sample

The population consisted of nurses working in Primary Health Care units in the State of São Paulo. The study sample for participant selection was based on convenience^11^, meeting the following inclusion criteria established for the study: female and male nurses, with no age limits, working in direct care or in the management of Basic Health Units (BHU) or Family Health Units (FHU) in the State of São Paulo. Nurses who had been working in nursing for more than one year and had no other occupation outside the nursing field, who had experienced musculoskeletal disorders in the last three months and had at least one episode lasting two hours or more^8^ were included. Nurses who had worked in another occupation outside the nursing field were excluded from the sample.

It should be noted that no prior sample calculation was performed, as this was a convenience sample based on participants’ availability and participation during the data collection period.

Invitations to participate in the study were sent by email by the Regional Nursing Council of São Paulo (COREN/SP).

### Study variables

The outcome variables evaluated in the study correspond to the total number of body locations at which the patient experiences the reported symptom. The symptoms evaluated were pain, numbness, fatigue, tingling, heaviness and other symptoms. The final classification of the Nurse-Work Instability Scale (Nurse-WIS), with responses low|medium, and high, was also evaluated as an outcome. The following were evaluated as independent variables of the study for all outcomes: age (in years); sex (male, female); family income (up to 3 minimum wages; above 3 to 7 minimum wages; above 7 minimum wages); body mass index (BMI) (kg/m²); physical exercise (yes, no); works as a nursing assistant/technical manager (yes, no); works as a primary care manager/supervisor (yes, no); type of contract (statutory, Consolidated Labor Laws - CLT, other); works in BHU/FHS (yes, no); length of time working in primary health care (years); length of time working as a nurse (years); weekly workload (up to 36 hours, 40 hours, over 40 hours); works morning/afternoon shift (yes, no), leave due to musculoskeletal symptoms (yes, no), leave due to anxiety (yes, no), leave due to d epression (yes, no).

### Data collection

Data collection was performed remotely using the Research Electronic Data Capture (REDCap) platform^13^, from July to September 2023. Nurses with active registration and valid e-mail addresses at COREN/SP were invited to participate in the study through a single invitation sent via institutional e-mail. The questionnaire remained open for responses from July 27 to September 30, 2023.

Two instruments were used: (1) a sociodemographic and occupational questionnaire, developed and composed of 22 items, nine of which were related to demographic characteristics and thirteen to occupational aspects, absenteeism and the presence of musculoskeletal symptoms, and (2) the Brazilian version of the Nurse Work Instability Scale (Nurse-WIS), translated and validated in Brazil^14^. The scale, consisting of 20 true/false statements, identifies the level of instability at work among professionals with musculoskeletal disorders, classifying the risk as low (<7 points), medium (7-13), or high (≥14 points). The Brazilian version had a reliability index of 0.931. The use of the Nurse-WIS scale was previously authorized by the copyright group linked to the University of Leeds, United Kingdom.

### Data analysis

The statistical analysis used GAMLSS (Generalized Additive Models for Location, Scale, and Shape), which allows parametric distributions of the response variable to be modeled using independent variables in a parametric or nonparametric manner^15^. This approach covers different types of distributions (continuous, discrete, asymmetric, or heavy-tailed), considering up to four parameters: position (μ), scale (σ), asymmetry (υ), and kurtosis (τ). Each parameter is associated with a set of independent variables through specific link functions. This approach provides a more comprehensive analysis that is better suited to the nature of the collected data.

The selection of the distribution of the response variable and independent variables was guided by Akaike’s information criterion (AIC)^16^, with standard penalty (k=2) for choosing outcomes, and k=4 for selecting predictors, as suggested in the literature. The BIC criterion (k=log(n)) was also considered^17^.

The adequacy of the models was verified using the Shapiro-Wilk normality test applied to the residuals. Measures of association such as relative increase (AR = (exp(β) - 1) × 100) and odds ratio were estimated. Correlations between numerical variables and outcomes were analyzed using Spearman’s coefficient, and comparisons between nominal variables and outcomes were performed using the Kruskal-Wallis, Pearson’s chi-square, and Fisher’s exact tests^18^.

The analyses were performed in R (version 4.3.1), at the 5% significance level (α = 0.05). The results were presented in tables.

### Ethical considerations

The study was conducted in accordance with national and international ethical guidelines and approved by the Research Ethics Committee of the Ribeirão Preto School of Nursing, University of São Paulo, under protocol CAAE: 57568822.0.0000.5393. Resolution 466/12 on ethical standards for research involving human subjects was followed^19^. All participants who consented to participate in the study signed the Informed Consent Form and their anonymity and data confidentiality were ensured.

## Results

A total of 286 nurses participated in the study; however, 143 (50.0%) presented work instability, with 14 (9.80%) presenting low risk, 47 (32.90%) medium risk and 82 (57.30%) high risk.

Among the nurses, 125 (87.40%) were women, 77 (54.60%) were married/lived with a partner, 99 (69.70%) were white, 68 (47.90%) had no children, 62 (43.70%) had an income between four and six minimum wages and 84 (58.70%) did not exercise regularly. The average age of the workers was 38.90 years, and the average BMI was 28.22 kg/m².

Regarding occupational data, 124 (86.70%) participants worked as nurses and 35 (24.50%) as primary care managers, 71 (50.00%) were employed under the statutory regime, 44 (34.60%) worked in the city of São Paulo, 70 (49.00%) worked in BHU, and 87 (60.80%) in FHU; the most prevalent weekly workload was 40 hours per week (104 - 73.20%), and 121 (84.60%) worked in the morning. Among nurses, 14 (9.80%) held another job, and most (7; 49.90%) worked as nursing assistants.

Among the 143 nurses who reported pain, 101 (70.60%) reported neck pain, 89 (62.20%) reported shoulder pain, 71 (49.70%) reported upper back pain, 11 (7.70%) reported elbow pain, 51 (35.70%) in the wrists/hands, 58 (40.60%) in the lower back, 27 (18.90%) in the hips/thighs, 43 (30.10%) in the knees, 32 (22.40%) in the ankles/feet, and nine (6.30%) in other areas.

Regarding tingling, 15 (10.50%) professionals reported it in the neck, 13 (9.10%) in the shoulders, four (2.80%) in the upper back and elbows respectively, 59 (41.30%) in the wrists/hands, nine (6.30%) in the upper back, seven (4.90%) in the hips/thighs, five (3.50%) in the knees, 31 (21.70%) in the ankles/feet, and eight (5.6%) reported it in another region.

Of the nurses who reported numbness in anatomical regions, 11 (7.70%) reported it in the neck, eight (5.60%) in the shoulders, four (2.80%) in the upper and lower back and knees, respectively, five (3.50%) in the elbows, 45 (31.50%) in the wrists/hands, six (4.20%) in the hips/thighs and another region, respectively, and 24 (16.80%) in the ankles/feet.

Regarding the feeling of heaviness in anatomical regions, 64 (44.80%) of nurses reported this symptom in the neck, 88 (61.50%) in the shoulders, 49 (34.30%) in the upper back, three (2.10%) in the elbows, ten (7.00%) in the wrists/hands, 31 (21.70%) in the lower back, 24 (16.80%) in the hips/thighs, 17 (11.90%) on the knees, 34 (23.80%) on the ankles/feet, and five (3.50%) on other parts of the body.

Regarding the presence of fatigue, 57 (39.90%) nurses reported it in the neck, 68 (47.60%) in the shoulders, 53 (37.10%) in the upper back, six (4.20%) in the elbows, 15 (10.50%) in the wrists/hands, 38 (26.60%) in the lower back, 24 (16.80%) in the hips/thighs, 21 (14.70%) in the knees, 31 (21.70%) in the ankles/feet, and 14 (9.80%) in another region.

Of the participants who reported the presence of another symptom, five (3.50%) reported it in the neck and lower back, respectively; seven (4.90%) in the shoulders and upper back, respectively; two (1.40%) in the elbows; one (0.70%) in the wrists/hands, four (2.8%) in the hips/thighs and ankles/feet, respectively; six (4.20%) in the knees and 12 (8.4%) in another region of the body. The symptoms reported were generally headache, convulsions, dyspnea, insomnia, tachycardia, dizziness, torticollis, vertigo, and precordialgia.

The data in Table 1 present the results of bivariate tests for sociodemographic and occupational characteristics and work instability among nurses. However, not all study participants answered all questionnaire items.

**Table 1:** Distribution of values obtained according to bivariate tests of sociodemographic and occupational characteristics and work instability among nurses working in Primary Health Care in São Paulo (n = 143). State of São Paulo, Brazil, 2023

	High	Low|Medium	Statistical test	p-value
**Total**	82	61		
**Sex**			Qui-Square (1 df*) = 0.12	0.730
Female	71 (56.8%)	54 (43.2%)		
Male	11 (61.1%)	7 (38.9%)		
**Family income** ^†^(minimum wage)			Qui-Square (1 df*) = 11.81	0.003
Between 1 and 3	12 (57.1%)	9 (42.9%)		
Between 4 and 6	26 (41.9%)	36 (58.1%)		
Above 7	43 (72.9%)	16 (27.1%)		
**Physical exercise**			Qui-Square (1 df*) = 0.4	0.529
No	50 (59.5%)	34 (40.5%)		
Yes	32 (54.2%)	27 (45.8%)		
**Care nurse**			Qui-Square (1 df*) = 0.2	0.658
No	10 (62.5%)	6 (37.5%)		
Yes	72 (56.7%)	55 (43.3%)		
**Primary care manager**			Chi-Square (1 df*) = 0.47	0.493
No	62 (59%)	43 (41%)		
Yes	20 (52.6%)	18 (47.4%)		
**Type of contract**			Fisher’s exact test	0.075
CLT^‡^	44 (67.7%)	21 (32.3%)		
Statutory	35 (49.3%)	36 (50.7%)		
Other	3 (50%)	3 (50%)		
BHU^§^			Chi-Square (1 df*) = 3.2	0.074
No	36 (50%)	36 (50%)		
Yes	46 (64.8%)	25 (35.2%)		
FHU^||^			Chi-Square (1 df*) = 1	0.317
No	35 (62.5%	21 (37.5%)		
Yes	47 (54%)	40 (46%)		
**Weekly workload (hours)**			Chi-Square (2 df*) = 0.36	0.835
Up to 36	17 (60.7%)	11 (39.3%)		
40	59 (56.7%)	45 (43.3%)		
Over 40	5 (50%)	5 (50%)		
**Work shift - morning**			Chi-Square (1 df*) = 0.08	0.773
No	12 (54.5%)	10 (45.5%)		
Yes	70 (57.9%)	51 (42.1%)		
**Work shift - afternoon**			Chi-Square (1 df*) = 0.16	0.690
No	14 (53.8%)	12 (46.2%)		
Yes	68 (58.1%)	49 (41.9%)		
**Anxiety**			Chi-Square (1 df*) = 0.47	0.495
No	74 (56.5%)	57 (43.5%)		
Yes	8 (66.7%)	4 (33.3%)		
**Depression**			Chi-Square (1 df*) = 0.01	0.942
No	75 (57.3%)	56 (42.7%)		
Yes	7 (58.3%)	5 (41.7%)		

*Df = Degrees of freedom; ^†^Family income = Current minimum wage of R$ 1,320.00, Brazil, 2023; ^‡^CLT = Consolidation of Labor Laws; ^§^UBS = Basic Health Unit; ^||^USF = Family Health Unit

For the analysis of the EIT classification data, the low and medium categories were grouped together due to the small number of respondents in the low work instability category. Among the nurses participating in the study, the majority reported high work instability.

Regarding the variables analyzed, there was an association between family income and work instability (p=0.003). Among participants, the majority reported high work instability and family income above 7 minimum wages (43-72.9%), indicating that higher income was associated with greater work instability.

Data regarding musculoskeletal symptoms, age, BMI, length of service in primary health care, and as a nursing assistant are presented in Table 2.

**Table 2:** Distribution of values obtained according to musculoskeletal symptoms, age (in years), body mass index (kg/m²), length of service in Primary Health Care and as a nurse (in years), and work instability among nurses working in Primary Health Care in São Paulo (n = 143). State of São Paulo, Brazil, 2023

Variables	WIS* classification	n	Average	Standard deviation	Minimum	Q^†^0.25	Q^†^0.5	Q^†^0.75	Maximum	p-value
Pain	High	82	3.72	1.57	1.00	3.00	3.00	5.00	8.00	0.0164
Pain	Low|Medium	61	3.07	1.72	0.00	2.00	3.00	4.00	9.00	
Numbness	High	82	0.95	1.15	0.00	0.00	1.00	1.75	7.00	0.0698
Numbness	Low|Medium	61	0.64	0.91	0.00	0.00	0.00	1.00	5.00	
Tingling	High	82	1.22	1.23	0.00	0.00	1.00	2.00	7.00	0.0639
Tingling	Low|Medium	61	0.90	1.03	0.00	0.00	1.00	1.00	5.00	
Heaviness	High	82	2.60	1.68	0.00	2.00	2.00	3.00	9.00	0.0023
Heaviness	Low|Medium	61	1.84	1.50	0.00	1.00	1.00	3.00	8.00	
Fatigue	High	82	2.66	2.41	0.00	1.00	2.00	4.00	10.00	0.0233
Fatigue	Low|Medium	61	1.79	1.92	0.00	0.00	1.00	3.00	9.00	
Other symptoms	High	82	0.43	0.86	0.00	0.00	0.00	1.00	5.00	0.2823
Other symptoms	Low|Medium	61	0.30	0.67	0.00	0.00	0.00	0.00	3.00	
Age	High	61	38.59	8.52	23.00	33.00	38.00	44.00	66.00	0.7547
Age	Low|Medium	80	39.14	7.13	26.00	34.00	38.50	43.00	60.00	
BMI^‡^	High	61	27.77	5.93	18.65	22.89	27.10	31.62	42.15	0.4439
BMI^‡^	Low|Medium	80	28.56	5.76	19.31	24.92	27.09	30.93	48.88	
Length of service in primary health care	High	61	9.23	7.00	0.00	3.00	9.00	13.00	40.00	0.9299
Length of service in primary health care	Low|Medium	82	9.20	6.32	1.00	4.25	8.00	12.75	33.00	
Length of service as a nurse	High	61	11.41	6.60	0.00	7.00	12.00	17.00	24.00	0.3838
Length of service as a nurse	Low|Medium	82	12.73	6.55	2.00	9.00	11.50	17.00	34.00	

*WIS = Work Instability Scale; ^†^Q = Quartile; ^‡^BMI = Body Mass Index

Statistical tests identified differences between pain and high instability at work (p=0.0164), feeling of heaviness and high instability at work (p=0.0023), and fatigue and high instability at work (p=0.0233).

Regarding pain, nurses reported an average of 3.72 areas of pain with high instability at work.

Regarding the feeling of heaviness, it was found that participants had an average of 2.60 regions with a feeling of heaviness in high instability at work.

Finally, there was also an average of 2.66 regions with fatigue in high work instability among participants. 

Table 3 presents the data from the regression analysis of instability at work in relation to pain, numbness, tingling, feeling of heaviness, fatigue, other symptoms, and family income. 

**Table 3:** Regression analysis of work instability among nurses working in Primary Health Care in São Paulo who experienced work instability (n = 143). State of São Paulo, Brazil, 2023

µ*	Estimate	Standard deviation	p-value	Pr(>|t|)^†^	Relative increase	Confidence Interval	
(Intercept)	-0.1169	0.5043	-0.2318	0.8171	OR^‡^	LI_OR^§^	LS_OR^||^
Pain	0.0956	0.1564	0.6110	0.5423	1.1003	0.8098	1.4950
Numbness	0.0506	0.2646	0.1912	0.8457	1.0519	0.6263	1.7667
Tingling	0.1281	0.2382	0.5378	0.5916	1.1367	0.7127	1.8129
Heaviness	0.2792	0.1966	1.4204	0.1579	1.3221	0.8994	1.9436
Fatigue	-0.0160	0.1280	-0.1250	0.9007	0.9841	0.7657	1.2648
Other symptoms	0.3329	0.2595	1.2832	0.2018	1.3951	0.8390	2.3198
Family income between 1 and 3 minimum wages	-1.2242	0.6027	-2.0310	0.0443	0.2940	0.0902	0.9581
Family income between 4 and 6 minimum wages	-1.4687	0.4216	-3.4832	0.0007	0.2302	0.1008	0.5261

*µ = Greek letter “mu” (population mean); ^†^Pr(>|t|) = p-value; ^‡^OR = Odds Ratio; ^§^LI_OR = Lower limit of the odds ratio confidence interval; ^||^LS_OR = Upper limit of the odds ratio confidence interval; ^¶^Family income = Current minimum wage of BRL 1,320.00, Brazil, 2023

Based on the regression analysis, participants with a family income between one and three minimum wages had an average score of 70.60% (1 - 0.2940) lower in work instability than those with an income above seven minimum wages (p=0.0443). In addition, nurses with a family income between four and six minimum wages demonstrated an average score of 76.98% (1 - 0.2302) (p=0.0007) lower in instability than those with an income above seven minimum wages.

Table 4, below, shows absenteeism among nurses in the last 12 months, manifested through symptoms such as instability at work.

**Table 4:** Distribution of tests obtained according to the presence of musculoskeletal symptoms and instability at work among nurses working in Primary Health Care in São Paulo (n = 143). State of São Paulo, Brazil, 2023

	High	Low| Medium	Statistical test	p-value
**Musculoskeletal symptoms in the last 12 months**			Chi-Square (1 df*) = 1.43	0.231
Yes	29 (65.9%)	15 (34.1%)		
No	53 (53.5%)	46 (46.5%)		

*Df = Degrees of freedom

Regarding the presence of musculoskeletal symptoms in the past 12 months and work instability, no statistically significant association was found; however, among the 29 (65.9%) nurses who took leave, the majority were at high risk for work instability.

## Discussion

The study found that most participants were female, married, white, childless, with an average age of 38.90 years and a monthly income between 4 and 6 minimum wages. In a survey conducted among nurses with work instability, it was evident that the study population was mostly female, confirming the predominance of women in nursing work. Regarding weight and BMI, the average was 77.38 kg and 28.22 kg/m², respectively^20^; in addition, most participants did not exercise regularly and were classified as overweight^3^.

Similar data regarding sex and age were found in a study that assessed work instability among nursing staff at a Brazilian university hospital^21^.

In terms of nursing work, most nurses worked in PHC, in the city of São Paulo, under the statutory regime. In São Paulo, most nurses worked in family health units during the morning and the most prevalent weekly workload was 40 hours. Still on occupational characteristics, a minority had other employment relationships. It was also found, that nurses had an average of 9.21 years of work in PHC and 12.17 years working as nurses.

Among the musculoskeletal symptoms reported by nurses with work instability, neck pain, tingling and numbness in the wrists/hands, heaviness, and shoulder fatigue were the most commonly reported. Other symptoms reported were body pain, irritability, body burning, headache, tachycardia, vertigo, torticollis, anxiety, convulsions, dyspnea, fatigue, insomnia, herpes zoster lesions, and low back pain.

No studies on work instability among PHC nurses were found; however, in a survey of nursing professionals at a university hospital in Brazil, it was found that the lower back, upper back, shoulders, ankles and feet, neck, and knees were the areas most affected by musculoskeletal disorders, in that order^21^.

This study found that there was an association between family income and work instability, and among nurses, the majority reported high work instability and family income above seven minimum wages. The regression analysis found that participants with a family income between 1 and 3 minimum wages had an average score 70.60% lower for work instability than those with an income above 7 minimum wages, and nurses with family incomes between 4 and 6 minimum wages showed an average score of 76.98% lower in instability than those with incomes above 7 minimum wages.

Through this, it can be stated that the higher the income, the greater the chance of work instability among nurses, and this situation may be caused by low nursing salaries and the need to hold multiple jobs to improve income. 

For several years, Brazilian nurses have sought recognition, rights and decent working conditions; however, they have faced numerous difficulties and obstacles^22^. In this context, the valuation and minimum wage for nurses involve historical and moral considerations, as well as the promotion of dignified and decent work for this professional class^23^.

High risk of work-related instability due to musculoskeletal symptoms has been identified in other studies of nursing professionals^19^ ^),(^ ^21^, which may be attributable to the types of work activities performed, work organization, and the presence of occupational hazards.

A literature review showed that musculoskeletal disorders in nurses were associated with physical load, organizational, and psychosocial conditions related to the socioeconomic context, increasing vulnerability to illness^24^ ^)-(^ ^25^.

With the analyses, statistical differences were identified between the symptoms of pain, heaviness, and fatigue associated with high instability at work. Regarding pain, nurses reported an average of 3.72 pain areas in high instability at work. Regarding the feeling of heaviness, participants reported an average of 2.60 regions of heaviness during periods of high work instability. Finally, an average of 2.66 regions reported fatigue among nurses with high work instability.

Based on these results, it can be stated that musculoskeletal symptoms (pain, heaviness and fatigue) and high instability can lead to discrepancies between nurses’ abilities and work activities, thereby interfering with work practices.

Regarding instability at work and musculoskeletal symptoms in the last 12 months, most nurses were identified as at high risk for instability, although there was no statistically significant association between these variables. However, the presence of musculoskeletal symptoms may be associated with a higher risk of work instability^7^ ^)-(^ ^8^ and, in addition, highlights the importance of further studies on the impact of work instability and absenteeism.

As limitations, the cross-sectional study does not establish a relationship between cause and effect of the variables studied; the use of a self-administered questionnaire may cause interference from uncontrolled factors and the inability to compare some results with other scientific studies related to work instability and musculoskeletal symptoms among PHC nurses. Convenience sampling limited the generalizability of the results, but it was adopted given the feasibility of participant access and the ease of workplace data collection, thereby enabling the collection of relevant data on work instability among primary health care nurses. In addition, self-reporting of symptoms over the past three months may be subject to memory bias, which can affect the accuracy of reported symptoms and introduce temporal limitations. Geographic restrictions limit the generalizability of results, as working conditions and employment relationships may vary across regions of the country.

However, this study made important advances by demonstrating the complex relationship between work instability and musculoskeletal disorders in PHC nurses in the state of São Paulo. The research deepened the understanding of individual and occupational factors that influence the prevalence and severity of musculoskeletal symptoms, such as pain, tingling, and heaviness, highlighting, for example, the influence of age, BMI, family income, and workload. In addition, the study highlighted that work instability is associated with distinct symptom profiles and that identification can be a strategic tool to prevent absenteeism, professional abandonment, and early retirement, supporting the development of policies and interventions that promote better working conditions and the health of nursing professionals.

## Conclusion

The study analyzed the incidence of work instability among nurses working in primary health care in the state of São Paulo, its association with musculoskeletal disorders and its impact on absenteeism.

Among musculoskeletal symptoms among nurses with work instability, the study found that neck pain, wrist/hand tingling and numbness, heaviness, and shoulder fatigue occurred most frequently.

Statistical analyses of nurses with work instability showed an association between family income and work instability.

Regarding musculoskeletal symptoms (pain, heaviness, and fatigue), differences were observed among individuals with high work instability.

This study concludes that musculoskeletal symptoms and work instability were correlated with individual conditions and behaviors and the working conditions of PHC nurses. Furthermore, early identification of work-related instability among this professional category can minimize reductions in work capacity and, consequently, prevent absenteeism, abandonment of the profession, and early retirement due to musculoskeletal symptoms. Identifying workplace instability is essential for guiding actions and strategies to improve the working conditions of nursing professionals, and to support occupational health prevention and promotion policies for nurses.

## Data Availability

All data generated or analysed during this study are included in this published article.
