# Supplementary material for: Work instability, musculoskeletal disorders and absenteeism among Primary Care nurses
Source: Rev Lat Am Enfermagem. 2026 Oct 7;34:e4939. doi: 10.1590/1518-8345.8307.4939 (PMC13679913; doi:10.1590/1518-8345.8307.4939)
Supplement: Translation

# Translation: Instabilidade no trabalho, distúrbios osteomusculares e absenteísmo em enfermeiros da Atenção Primária*

**DOI:** 10.1590/1518-8345.8307.4940

**Authors:** Heloisa Ehmke Cardoso dos Santos, Rafael de Souza Petersen, Fernanda Ludmilla Rossi Rocha, Lacir José Santin, Maria Helena Palucci Marziale

**Affiliations:** 1 Universidade de São Paulo, Escola de Enfermagem de Ribeirão Preto, Centro Colaborador da OPAS/OMS para o Desenvolvimento da Pesquisa em Enfermagem, Ribeirão Preto, SP, Brasil.; 2 Fundação Oswaldo Cruz, Brasília, DF, Brasil.

**Keywords:** Segurança do Emprego, Transtornos Traumáticos Cumulativos, Atenção Primária à Saúde, Enfermagem, Saúde Ocupacional, Absenteísmo

## Abstract

**Objetivo:**

analisar a associação entre instabilidade no trabalho, distúrbios osteomusculares e absenteísmo em enfermeiros da Atenção Primária à Saúde.

**Método:**

estudo metodológico, transversal, quantitativo, realizado com 286 enfermeiros(as) da Atenção Primária à Saúde. Utilizaram-se o Questionário Sociodemográfico e Ocupacional e a versão brasileira da *Nurse-Work Instability Scale*. A coleta de dados ocorreu via plataforma digital, por meio de estatística descritiva, testes estatísticos e modelos de regressão (p<0,05). Para a elaboração do estudo foi utilizado o *Strengthening the Reporting of Observational studies in Epidemiology* (STROBE).

**Resultados:**

participaram do estudo 286 enfermeiros, contudo 143 (50,0%) apresentaram instabilidade no trabalho, sendo 9,8% de baixo risco, 32,9% de médio risco e 57,3% de alto risco. Houve associação significativa entre alta instabilidade e sintomas musculoesqueléticos como dor, sensação de peso e fadiga, afetando múltiplas regiões corporais. Notou-se maior instabilidade entre profissionais com maior renda familiar. Os distúrbios musculoesqueléticos foram as principais causas de absenteísmo, embora não tenha havido associação estatística entre instabilidade e afastamentos.

**Conclusão:**

a instabilidade no trabalho entre enfermeiros(as) da Atenção Primária à Saúde foi prevalente e relacionada aos fatores ocupacionais e físicos, exigindo ações para preservação da capacidade laboral.

## Destaques

**(1)** Estudo analisou instabilidade no trabalho e dores musculoesqueléticas na Atenção Primária à Saúde.

**(2)** Instabilidade associou-se à dor no pescoço e maior absenteísmo nos enfermeiros.

**(3)** Alta instabilidade associou-se à dor, peso e fadiga em várias regiões do corpo.

**(4)** Sintomas musculoesqueléticos foram as principais causas de afastamento por doença.

**(5)** Resultados impactam políticas de saúde e gestão do trabalho na enfermagem.

## Introdução

O trabalho humano é um dos principais motores para o desenvolvimento social e econômico, proporcionando ao indivíduo não apenas sustento, mas também importantes relações sociais e oportunidades de crescimento profissional. Nesse contexto, a saúde e a segurança no trabalho, bem como a capacidade funcional dos trabalhadores, são determinantes para a sustentabilidade dos sistemas produtivos e para o bem-estar social^1^.

A enfermagem, enquanto profissão central na área da saúde, tem papel fundamental na promoção da qualidade de vida e no cuidado integral às pessoas, atuando em diferentes níveis do sistema de saúde, especialmente na Atenção Primária à Saúde (APS)^2^.

No Brasil, os profissionais de enfermagem representam a segunda maior categoria de trabalhadores de nível superior na saúde, sendo essenciais para a operacionalização das políticas públicas e para a garantia do acesso universal e integral aos serviços de saúde^3^. Contudo, esses profissionais frequentemente enfrentam sobrecarga de trabalho, múltiplas funções e exigências crescentes, fatores que elevam o risco de adoecimento físico e mental, especialmente na APS, onde as condições de trabalho e os desafios organizacionais impactam diretamente sua saúde^4^. Além disso, um estudo internacional mostrou que fatores como jornadas prolongadas, maior carga física e diminuição da autonomia do profissional podem provocar maior vulnerabilidade para o surgimento dos distúrbios osteomusculares, evidenciando a influência do contexto socioeconômico na ocorrência desses distúrbios^5^. 

Os riscos ocupacionais na enfermagem abrangem desde fatores físicos, químicos e biológicos até riscos ergonômicos e psicossociais. Destacam-se, especialmente, os distúrbios osteomusculares relacionados ao trabalho (DORT), ocasionados por posturas inadequadas, esforços repetitivos e longas jornadas, que resultam em limitações funcionais e impacto na capacidade para o trabalho^6^. Paralelamente, os riscos psicossociais, como o estresse laboral, assédio, insegurança e falta de suporte, têm sido reconhecidos como importantes fatores de adoecimento mental entre enfermeiros(as), contribuindo para a síndrome de *burnout*, ansiedade e depressão^7^.

No ambiente laboral, o período antes da deficiência para o trabalho pode ocorrer quando o trabalhador começa a apresentar dificuldades para cumprir as atividades laborais, sendo necessárias intervenções neste momento para prevenir o afastamento e até a perda do emprego^8^ ^)-(^ ^9^. Com isso, a instabilidade no trabalho pode ser vista como um estado transitório e potencialmente reversível se for identificada e tratada precocemente, promovendo benefícios nos âmbitos psicossociais e econômicos para o(a) trabalhador(a) e instituição empregadora^9^, minimizando os afastamentos e a redução da capacidade no trabalho^10^. 

Diante da relevância da enfermagem na APS e dos impactos negativos das condições laborais sobre a saúde e produtividade desses profissionais, torna-se fundamental investigar a instabilidade no trabalho, especialmente no que tange aos distúrbios osteomusculares e absenteísmo. Embora existam estudos sobre riscos ocupacionais e distúrbios osteomusculares em enfermeiros, a instabilidade no trabalho ainda é um tema pouco explorado, sobretudo na APS. Essa lacuna de conhecimento limita a compreensão sobre como os vínculos laborais precários e condições de trabalho instáveis podem afetar a saúde física e mental desses profissionais, repercutindo na capacidade para o trabalho e no absenteísmo.

Assim, este estudo teve como objetivo analisar a associação entre instabilidade no trabalho, distúrbios osteomusculares e absenteísmo em enfermeiros da Atenção Primária à Saúde.

## Método

### Delineamento do estudo

Trata-se de um estudo realizado em duas etapas procedimentais: a primeira etapa compreendeu um estudo metodológico de construção e validação de um questionário e a segunda de um estudo observacional, descritivo, transversal, com abordagem quantitativa dos dados e para a elaboração do estudo foi utilizado o *Strengthening the Reporting of Observational Studies in Epidemiology* (STROBE).

A primeira etapa correspondeu à construção e validação de um questionário sociodemográfico e ocupacional para enfermeiros(as) da Atenção Primária à Saúde, fundamentada nos referenciais de Polit e Beck^11^ e Pasquali^12^. Inicialmente, elaborou-se a versão preliminar do instrumento com base na revisão de literatura e princípios teóricos de formulação de itens, estrutura e semântica. Em seguida, o instrumento foi submetido à validação de conteúdo por um comitê de sete especialistas com experiência em saúde ocupacional, distúrbios musculoesqueléticos e saúde mental. A validação ocorreu em três rodadas, com análise da relevância dos itens e cálculo do Índice de Validade de Conteúdo (IVC), obtendo-se IVC final de 1,0. Posteriormente, foi realizado o pré-teste com 30 enfermeiros(as) da APS para verificar a clareza, aplicabilidade e equivalência semântica e cultural do questionário sociodemográfico e ocupacional. Os participantes do pré-teste foram incluídos na amostra final do estudo e os resultados confirmaram a adequação do questionário para sua aplicação em larga escala. A segunda etapa consistiu em um estudo observacional, descritivo e transversal, com abordagem quantitativa dos dados em que foram utilizados os seguintes questionários para a coleta de dados: questionário sociodemográfico e ocupacional (Quest APS - sociodemográfico ocupacional) e Escala de Instabilidade no Trabalho de Enfermagem (EIT- enfermagem)

### Local do estudo

Unidades de Atenção Primária à Saúde do Estado de São Paulo, Brasil, no período de 27 de julho de 2023 a 30 de setembro de 2023.

### População e amostra

A população foi constituída pelos enfermeiros(as) atuantes em unidades de Atenção Primária à Saúde do Estado de São Paulo. A amostragem do estudo para a seleção dos participantes foi obtida por conveniência^11^, atendendo aos seguintes critérios de inclusão estabelecidos para o estudo: enfermeiros(as) do sexo feminino e masculino, sem limites de idades, atuantes na assistência direta ou na gestão de Unidades Básicas de Saúde (UBS) ou Unidades de Saúde da Família (USF) no Estado de São Paulo. Foram incluídos enfermeiros (as) que exerceram atividade na enfermagem há mais de um ano e sem outra ocupação fora da área da enfermagem, que apresentaram distúrbio osteomuscular nos últimos três meses e com pelo menos um episódio com duração igual ou maior que duas horas^8^. Os(as) enfermeiros (as) que exerceram outra ocupação fora da área da enfermagem foram excluídos da amostra.

Destaca-se que não foi realizado cálculo amostral prévio, uma vez que se tratou de amostragem por conveniência, baseada na disponibilidade e adesão dos participantes no período de coleta de dados.

Os convites para participação no estudo foram enviados, via e-mail, pelo Conselho Regional de Enfermagem de São Paulo (COREN/SP).

### Variáveis do estudo

As variáveis de desfecho do estudo avaliadas correspondem ao número total de locais do corpo nos quais os(as) enfermeiros(as) manifestaram o sintoma referido. Os sintomas avaliados foram dor, dormência, fadiga, formigamento, sensação de peso e outro sintoma. Também foi avaliado como desfecho a classificação final da Escala de Instabilidade do Trabalho (EIT-Enfermagem), com as respostas baixo|médio e alto. Como variáveis independentes do estudo para todos os desfechos foram avaliadas: idade (em anos); sexo (masculino, feminino); renda familiar (até 3 salários-mínimos; acima de 3 até 7 salários-mínimos; acima de 7 salários-mínimos); índice de massa corporal (IMC) (kg/m²); prática de exercício físico (sim, não); trabalha como enfermeiro assistencial/responsável técnico (sim, não); trabalha como gerente/supervisor de atenção primária (sim, não); tipo de contrato (estatutário, Consolidação das Leis do Trabalho - CLT, outro); trabalha em Unidade Básica de Saúde/Unidade de Saúde da Família - UBS/USF (sim, não); tempo de trabalho na Atenção Primária a Saúde (anos); tempo de profissão de enfermeiro (anos); carga horária semanal (até 36 horas, 40 horas, acima 40 horas); trabalha no turno da manhã/tarde (sim, não), afastamento por sintomas musculoesqueléticos (sim, não), afastamento por motivo de ansiedade (sim, não), afastamento por motivo de depressão (sim, não).

### Coleta dos dados

A coleta de dados foi realizada de forma remota por meio da plataforma *Research Electronic Data Capture* (REDCap)^13^, durante os meses de julho a setembro de 2023. Enfermeiros(as) com registro ativo e e-mails válidos no COREN/SP foram convidados a participar do estudo por meio de um único envio de convite: via e-mail institucional. O questionário permaneceu disponível para resposta entre 27 de julho e 30 de setembro de 2023.

Foram utilizados dois instrumentos: (1) um questionário sociodemográfico e ocupacional, elaborado e composto por 22 itens, sendo nove relacionados às características demográficas e treze aos aspectos ocupacionais, absenteísmo e presença de sintomas musculoesqueléticos e (2) a versão nacional da escala *Nurse Work Instability Scale* (Nurse-WIS), traduzida e validada no Brasil^14^. A escala, composta por 20 afirmações do tipo verdadeiro/falso, identifica o nível de instabilidade no trabalho entre profissionais com distúrbios musculoesqueléticos, classificando o risco como baixo (<7 pontos), médio (7-13) ou alto (≥14 pontos). A versão brasileira apresentou índice de confiabilidade de 0,931. O uso da escala Nurse-WIS foi previamente autorizado pelo grupo dos direitos autorais, vinculado à Universidade de Leeds, Reino Unido.

### Análise dos dados

A análise estatística utilizou modelos da classe GAMLSS (*Generalized Additive Models for Location, Scale and Shape*), que permitem modelar distribuições paramétricas da variável resposta com variáveis independentes de forma paramétrica ou não paramétrica^15^. Essa abordagem abrange diferentes tipos de distribuições (contínuas, discretas, assimétricas ou com caudas pesadas), considerando até quatro parâmetros: posição (μ), escala (σ), assimetria (υ) e curtose (τ). Cada parâmetro é associado a um conjunto de variáveis independentes, por meio de funções de ligação específicas. Essa abordagem oferece uma análise mais completa e ajustada à natureza dos dados coletados.

A seleção da distribuição da variável resposta e das variáveis independentes foi guiada pelo critério de informação de Akaike (AIC)^16^, com penalidade-padrão (k=2) para escolha dos desfechos e k=4 para seleção de preditores, conforme sugerido na literatura. O critério BIC (k=log(n)) também foi considerado^17^.

A adequação dos modelos foi verificada por meio do teste de normalidade de Shapiro-Wilk aplicado aos resíduos. Foram estimadas medidas de associação como aumento relativo (AR = (exp(β) - 1) × 100) e *odds ratio*. As correlações entre variáveis numéricas e desfechos foram analisadas pelo coeficiente de Spearman e a comparação entre variáveis nominais e os desfechos foi realizada com os testes de Kruskal-Wallis, qui-quadrado de Pearson e exato de Fisher^18^.

As análises foram conduzidas no *software* R (versão 4.3.1), adotando-se nível de significância de 5% (α = 0,05). Os resultados foram apresentados em tabelas.

### Considerações éticas

O estudo foi conduzido de acordo com as diretrizes de ética nacionais e internacionais e aprovado pelo Comitê de Ética em Pesquisa da Escola de Enfermagem de Ribeirão Preto da Universidade de São Paulo sob protocolo CAAE: 57568822.0.0000.5393. A Resolução 466/12 referente às normas éticas de pesquisas envolvendo seres humanos foi seguida^19^. Todos os participantes que consentiram em participar do estudo assinaram o Termo de Consentimento Livre e Esclarecido (TCLE) e sua anonimização e a confidencialidade dos dados foram asseguradas. 

## Resultados

Participaram do estudo 286 enfermeiros; contudo, 143 (50,0%) apresentaram instabilidade no trabalho, sendo que 14 (9,80%) apresentaram baixo risco, 47 (32,90%) médio risco e 82 (57,30%) alto risco. 

Dentre os(as) enfermeiros(as), 125 (87,40%) eram mulheres, 77 (54,60%) casados/viviam com companheiro, 99 (69,70%) brancos, 68 (47,90%) não possuíam filhos, 62 (43,70%) tinham uma renda entre quatro e seis salários mínimos e 84 (58,70%) não praticavam exercício físico regularmente. A idade média dos trabalhadores foi de 38,90 anos e a média do IMC foi de 28,22 kg/m².

Com relação aos dados ocupacionais, 124 (86,70%) participantes atuavam como enfermeiros(as) assistenciais e 35 (24,50%) como gerentes de atenção primária, 71 (50,00%) eram contratados pelo regime estatutário, 44 (34,60%) trabalhavam na cidade de São Paulo, 70 (49,00%) atuavam em UBS e 87 (60,80%) em USF; a carga horária semanal mais prevalente foi a de 40 horas semanais (104 - 73,20%) e 121 (84,60%) atuavam no período da manhã. Dos enfermeiros(as), 14 (9,80%) tinham outro vínculo empregatício e a maioria (7 - 49,90%) trabalhava como enfermeiro(a) assistencial. 

Dentre os 143 enfermeiros (as) que informaram a presença da dor, 101 (70,60%) referiram-na no pescoço, 89 (62,20%) nos ombros, 71 (49,70%) na parte superior das costas, 11 (7,70%) nos cotovelos, 51 (35,70%) nos punhos/mãos, 58 (40,60%) na parte inferior das costas, 27 (18,90%) no quadril/coxas, 43 (30,10%) nos joelhos, 32 (22,40%) nos tornozelos/pés, nove (6,30%) em outra região.

Em relação ao formigamento, 15 (10,50%) profissionais referiram-no no pescoço, 13 (9,10%) nos ombros, quatro (2,80%) na parte superior das costas e cotovelos respectivamente, 59 (41,30%) nos punhos/mãos, nove (6,30%) na parte superior das costas, sete (4,90%) no quadril/coxas, cinco (3,50%) nos joelhos, 31 (21,70%) nos tornozelos/pés e oito (5,6%) relataram-no em outra região. 

Dos enfermeiros(as) que manifestaram a dormência nas regiões anatômicas, 11 (7,70%) apresentaram-na no pescoço, oito (5,60%) nos ombros, quatro (2,80%) na parte superior e inferior das costas e joelhos respectivamente, cinco (3,50%) nos cotovelos, 45 (31,50%) nos punhos/mãos, seis (4,20%) no quadril/coxas e em outra região respectivamente e 24 (16,80%) nos tornozelos/pés.

Em relação à sensação de peso nas regiões anatômicas, 64 (44,80%) dos enfermeiros(as) relataram este sintoma no pescoço, 88 (61,50%) nos ombros, 49 (34,30%) na parte superior das costas, três (2,10%) nos cotovelos, dez (7,00%) nos punhos/mãos, 31 (21,70%) na parte inferior das costas, 24 (16,80%) no quadril/coxas, 17 (11,90%) nos joelhos, 34 (23,80%) nos tornozelos/pés e cinco (3,50%) em outro local do corpo. 

Em relação à presença da fadiga, 57 (39,90%) enfermeiros(as) referiram no pescoço, 68 (47,60%) nos ombros, 53 (37,10%) na parte superior das costas, seis (4,20%) nos cotovelos, 15 (10,50%) nos punhos/mãos, 38 (26,60%) na parte inferior das costas, 24 (16,80%) no quadril/coxas, 21 (14,70%) nos joelhos, 31 (21,70%) nos tornozelos/pés e 14 (9,80%) em outra região.

Dos participantes que referiram a presença de outro sintoma, cinco (3,50%) referiram no pescoço e parte inferior das costas, respectivamente; sete (4,90%) nos ombros e parte superior das costas, respectivamente; dois (1,40%) nos cotovelos; um (0,70%) nos punhos/mãos, quatro (2,8%) no quadril/coxas e tornozelos/pés, respectivamente; seis (4,20%) nos joelhos e 12 (8,4%) em outra região do corpo. Os sintomas relatados, de um modo geral, foram: cefaleia, convulsão, dispneia, insônia, taquicardia, tontura, torcicolo, vertigem, precordialgia. 

Os dados da Tabela 1 mostram os valores dos testes bivariados das características sociodemográficas, ocupacionais e instabilidade no trabalho de enfermeiros(as). Contudo, destaca-se que nem todos os participantes do estudo responderam a todas as perguntas do questionário.

**Tabela 1:** Distribuição dos valores obtidos segundo testes bivariados das características sociodemográficas, ocupacionais e resultado da instabilidade no trabalho dos(as) enfermeiros(as) atuantes na Atenção Primária à Saúde paulista (n = 143). Estado de São Paulo, Brasil, 2023

	Alto	Baixo|Médio	Teste estatístico	Valor de p
**Total**	82	61		
**Sexo**			Qui-Quadrado (1 df*) = 0,12	0,730
Feminino	71 (56,8%)	54 (43,2%)		
Masculino	11 (61,1%)	7 (38,9%)		
**Renda familiar** ^†^ (salário-mínimo)			Qui-Quadrado (1 df*) = 11,81	0,003
Entre 1 e 3	12 (57,1%)	9 (42,9%)		
Entre 4 e 6	26 (41,9%)	36 (58,1%)		
Acima de 7	43 (72,9%)	16 (27,1%)		
**Prática de exercício físico**			Qui-Quadrado (1 df*) = 0,4	0,529
Não	50 (59,5%)	34 (40,5%)		
Sim	32 (54,2%)	27 (45,8%)		
**Enfermeiro(a) assistencial**			Qui-Quadrado (1 df*) = 0,2	0,658
Não	10 (62,5%)	6 (37,5%)		
Sim	72 (56,7%)	55 (43,3%)		
**Gerente de atenção primária**			Qui-Quadrado (1 df*) = 0,47	0,493
Não	62 (59%)	43 (41%)		
Sim	20 (52,6%)	18 (47,4%)		
**Tipo de contrato**			Exato de Fisher	0,075
CLT^‡^	44 (67,7%)	21 (32,3%)		
Estatutário	35 (49,3%)	36 (50,7%)		
Outro	3 (50%)	3 (50%)		
UBS^§^			Qui-Quadrado (1 df*) = 3,2	0,074
Não	36 (50%)	36 (50%)		
Sim	46 (64,8%)	25 (35,2%)		
USF^||^			Qui-Quadrado (1 df*) = 1	0,317
Não	35 (62,5%)	21 (37,5%)		
Até 36	17 (60,7%)	11 (39,3%)		
40	59 (56,7%)	45 (43,3%)		
Acima de 40	5 (50%)	5 (50%)		
**Turno de trabalho - manhã**			Qui-Quadrado (1 df*) = 0,08	0,773
Não	12 (54,5%)	10 (45,5%)		
Sim	70 (57,9%)	51 (42,1%)		
**Turno de trabalho - tarde**			Qui-Quadrado (1 df*) = 0,16	0,690
Não	14 (53,8%)	12 (46,2%)		
Sim	68 (58,1%)	49 (41,9%)		
**Ansiedade**			Qui-Quadrado (1 df*) = 0,47	0,495
Não	74 (56,5%)	57 (43,5%)		
Sim	8 (66,7%)	4 (33,3%)		
**Depressão**			Qui-Quadrado (1 df*) = 0,01	0,942
Não	75 (57,3%)	56 (42,7%)		
Sim	7 (58,3%)	5 (41,7%)		

*Df = *Degrees of freedom* (graus de liberdade); ^†^Renda familiar = Salário mínimo vigente de R$ 1.320,00, Brasil, 2023; ^‡^CLT = Consolidação das Leis do Trabalho; ^§^UBS = Unidade Básica de Saúde; ^||^USF = Unidade de Saúde da Família

Para a análise dos dados da classificação da EIT, foi realizado um agrupamento entre as categorias baixo e médio, devido a um pequeno número de respondentes na categoria baixa instabilidade no trabalho. Dentre os enfermeiros(as) participantes do estudo, a maioria apresentou alta instabilidade no trabalho. 

Com relação às variáveis analisadas, houve associação entre renda familiar e instabilidade no trabalho (p=0,003) e, dentre os participantes, a maioria apresentou alta instabilidade no trabalho e renda familiar acima de 7 salários mínimos (43-72,9%), o que mostrou que quanto maior foi a renda, maior a instabilidade no trabalho. 

Dados referentes aos sintomas musculoesqueléticos, idade, IMC, tempo de atuação na Atenção Primária à Saúde e como enfermeiro(a) assistencial são apresentados na Tabela 2.

**Tabela 2:** Distribuição dos valores obtidos segundo os sintomas musculoesqueléticos, idade (em anos), índice de massa corporal (kg/m²), tempo de atuação na Atenção Primária à Saúde e como enfermeiro(a) assistencial (em anos) e instabilidade no trabalho dos(as) enfermeiros(as) atuantes na Atenção Primária à Saúde paulista (n = 143). Estado de São Paulo, Brasil, 2023

Variáveis	Classificação EIT*	n	Média	Desvio-padrão	Mínimo	Q^†^0.25	Q^†^0.5	Q^†^0.75	Máximo	Valor de p
Dor	Alto	82	3,72	1,57	1,00	3,00	3,00	5,00	8,00	0,0164
Dor	Baixo|Médio	61	3,07	1,72	0,00	2,00	3,00	4,00	9,00	
Dormência	Alto	82	0,95	1,15	0,00	0,00	1,00	1,75	7,00	0,0698
Dormência	Baixo|Médio	61	0,64	0,91	0,00	0,00	0,00	1,00	5,00	
Formigamento	Alto	82	1,22	1,23	0,00	0,00	1,00	2,00	7,00	0,0639
Formigamento	Baixo|Médio	61	0,90	1,03	0,00	0,00	1,00	1,00	5,00	
Sensação de peso	Alto	82	2,60	1,68	0,00	2,00	2,00	3,00	9,00	0,0023
Sensação de peso	Baixo|Médio	61	1,84	1,50	0,00	1,00	1,00	3,00	8,00	
Fadiga	Alto	82	2,66	2,41	0,00	1,00	2,00	4,00	10,00	0,0233
Fadiga	Baixo|Médio	61	1,79	1,92	0,00	0,00	1,00	3,00	9,00	
Outro sintoma	Alto	82	0,43	0,86	0,00	0,00	0,00	1,00	5,00	0,2823
Outro sintoma	Baixo|Médio	61	0,30	0,67	0,00	0,00	0,00	0,00	3,00	
Idade	Alto	61	38,59	8,52	23,00	33,00	38,00	44,00	66,00	0,7547
Idade	Baixo|Médio	80	39,14	7,13	26,00	34,00	38,50	43,00	60,00	
IMC^‡^	Alto	61	27,77	5,93	18,65	22,89	27,10	31,62	42,15	0,4439
IMC^‡^	Baixo|Médio	80	28,56	5,76	19,31	24,92	27,09	30,93	48,88	
Tempo de atuação na atenção primária à saúde	Alto	61	9,23	7,00	0,00	3,00	9,00	13,00	40,00	0,9299
Tempo de atuação na atenção primária à saúde	Baixo|Médio	82	9,20	6,32	1,00	4,25	8,00	12,75	33,00	
Tempo de atuação como enfermeiro assistencial	Alto	61	11,41	6,60	0,00	7,00	12,00	17,00	24,00	0,3838
Tempo de atuação como enfermeiro assistencial	Baixo|Médio	82	12,73	6,55	2,00	9,00	11,50	17,00	34,00	

*EIT = Escala de Instabilidade no Trabalho; ^†^Q = Quartil; ^‡^IMC = Índice de massa corporal

Por meio dos testes estatísticos, identificou-se que ocorreram diferenças entre a dor e alta instabilidade no trabalho (p=0,0164), sensação de peso e alta instabilidade no trabalho (p=0,0023) e fadiga e alta instabilidade no trabalho (p=0,0233). 

Com relação à dor, os(as) enfermeiros(as) apresentaram um número médio de 3,72 regiões com dor na alta instabilidade no trabalho. 

Sobre a sensação de peso, constatou-se que os participantes tiveram um número médio de 2,60 regiões com sensação de peso na alta instabilidade no trabalho.

Por fim, houve também um número médio de 2,66 regiões com fadiga na alta instabilidade no trabalho entre os participantes. 

A Tabela 3 apresenta os dados da análise de regressão da instabilidade no trabalho em relação à dor, dormência, formigamento, sensação de peso, fadiga, outro sintoma e renda familiar. 

**Tabela 3:** Análise de regressão da instabilidade no trabalho entre os(as) enfermeiros(as) atuantes na Atenção Primária à Saúde paulista e que apresentaram instabilidade no trabalho (n = 143). Estado de São Paulo, Brasil, 2023

µ*	Estimativa	Desvio-padrão	Valor de p	Pr(>|t|)^†^	Aumento relativo	Intervalo de Confiança	
(Intercepto)	-0,1169	0,5043	-0,2318	0,8171	OR^‡^	LI_OR^§^	LS_OR^||^
Dor	0,0956	0,1564	0,6110	0,5423	1,1003	0,8098	1,4950
Dormência	0,0506	0,2646	0,1912	0,8457	1,0519	0,6263	1,7667
Formigamento	0,1281	0,2382	0,5378	0,5916	1,1367	0,7127	1,8129
Sensação de peso	0,2792	0,1966	1,4204	0,1579	1,3221	0,8994	1,9436
Fadiga	-0,0160	0,1280	-0,1250	0,9007	0,9841	0,7657	1,2648
Outro sintoma	0,3329	0,2595	1,2832	0,2018	1,3951	0,8390	2,3198
Renda familiar^¶^ entre 1 e 3 salários mínimos	-1,2242	0,6027	-2,0310	0,0443	0,2940	0,0902	0,9581
Renda familiar^¶^ entre 4 e 6 salários mínimos	-1,4687	0,4216	-3,4832	0,0007	0,2302	0,1008	0,5261

*µ = Letra grega “mi” (média populacional); ^†^Pr(>|t|) = Valor de p; ^‡^OR = *Odds Ratio* (razão de chances); ^§^LI_OR = Limite inferior do intervalo de confiança do *odds ratio*; ^||^LS_OR = Limite superior do intervalo de confiança do *odds ratio*; ^¶^Renda familiar = Salário mínimo vigente de R$ 1.320,00, Brasil, 2023

Com base na análise de regressão realizada, identificou-se que os participantes que possuíam renda familiar entre um e três salários-mínimos apresentaram uma pontuação média 70,60% (1 - 0,2940) (p=0,0443) menor de instabilidade no trabalho do que aqueles que possuíam renda superior a sete salários-mínimos. Além disso, os enfermeiros(as) com renda familiar entre quatro e seis salários-mínimos demonstraram uma pontuação média 76,98% (1 - 0,2302) (p=0,0007) menor na instabilidade do que aqueles com renda superior a sete salários-mínimos. 

A Tabela 4, a seguir, evidencia o absenteísmo entre os enfermeiros(as) nos últimos 12 meses, manifestado por meio de sintomas como instabilidade no trabalho.

**Tabela 4:** Distribuição dos testes obtidos segundo a presença de sintomas musculoesqueléticos e instabilidade no trabalho dos(as) enfermeiros(as) atuantes na Atenção Primária à Saúde paulista (n = 143). Estado de São Paulo, Brasil, 2023

	Alto	Baixo |Médio	Teste estatístico	Valor de p
**Sintomas musculoesqueléticos nos últimos 12 meses**			Qui-Quadrado (1 df*) = 1,43	0,231
Sim	29 (65,9%)	15 (34,1%)		
Não	53 (53,5%)	46 (46,5%)		

*Df = *Degrees of freedom* (graus de liberdade)

Com relação à presença de sintomas musculoesqueléticos nos últimos 12 meses e à instabilidade no trabalho, constatou-se que não houve significância por meio do teste estatístico; contudo, dos 29 (65,9%) enfermeiros(as) que se afastaram, a maioria apresentou alto risco para a instabilidade no trabalho. 

## Discussão

Por meio do estudo, constatou-se que a maioria dos participantes era do sexo feminino, casada, branca, sem filhos, com uma média de idade de 38,90 anos e com renda mensal entre 4 e 6 salários-mínimos. Em uma pesquisa realizada entre enfermeiros(as) com instabilidade no trabalho, evidenciou-se que a população do estudo, na sua maioria, era de mulheres, confirmando a predominância do sexo feminino no trabalho de enfermagem. Em relação ao peso e IMC, a média foi de 77,38 kg e 28,22 kg/m², respectivamente^20^; ademais, a maior parte dos participantes não praticava exercício físico com regularidade, sendo classificada com sobrepeso^3^.

Dados similares em relação ao sexo e idade foram encontrados em um estudo que avaliou a instabilidade no trabalho entre trabalhadores de enfermagem de um hospital universitário brasileiro^21^.

Na atuação do trabalho de enfermagem, a maioria dos enfermeiros(as) trabalhava na assistência da APS, na cidade de São Paulo e pelo regime estatutário. Já sobre o local de trabalho, a maior parte de enfermeiros(as) paulistanos atuava em USF, no período da manhã e a carga horária semanal mais prevalente foi a de 40 horas semanais. Ainda sobre as características ocupacionais, a minoria possuía outro vínculo empregatício. Constatou-se, também, que os(as) enfermeiros(as) apresentavam em média 9,21 anos de trabalho na APS e 12,17 anos trabalhando como enfermeiro(a). 

Dentre os sintomas musculoesqueléticos dos(as) enfermeiros(as) com instabilidade no trabalho, a dor no pescoço, o formigamento e a dormência nos punhos/mãos, a sensação de peso e a fadiga nos ombros foram os mais relatados. Os outros sintomas referidos foram dor no corpo, irritabilidade, ardência corporal, cefaleia, taquicardia, vertigem, torcicolo, ansiedade, convulsão, dispneia, fadiga, insônia, lesão por herpes-zoster e lombalgia.

Estudos sobre a instabilidade no trabalho entre enfermeiros(as) da APS não foram encontrados; todavia, em uma pesquisa realizada com profissionais de enfermagem de um hospital universitário do Brasil, constatou-se que a região lombar, parte superior das costas, ombros, tornozelos e pés, pescoço e joelhos foram as regiões mais acometidas por distúrbios osteomusculares, nessa ordem^21^. 

Neste estudo foi constatado que houve associação entre renda familiar e instabilidade no trabalho e, dentre os(as) enfermeiros(as), a maioria apresentou alta instabilidade no trabalho e renda familiar acima de 7 salários-mínimos. A análise de regressão realizada constatou que os participantes que possuíam renda familiar entre 1 e 3 salários-mínimos apresentaram uma pontuação média 70,60% menor de instabilidade no trabalho do que os que possuíam renda superior a 7 salários-mínimos e, aqueles com renda familiar entre 4 e 6 salários-mínimos demonstraram uma pontuação média de 76,98% menor na instabilidade do que os que tinham renda superior a 7 salários-mínimos. 

Estes dados permitem afirmar que quanto maior for a renda, maior a chance de ocorrência da instabilidade no trabalho entre enfermeiros(as) e essa situação pode ser ocasionada pelo baixo salário da enfermagem e a necessidade de possuir mais de um emprego a fim de melhorar a renda. 

Há alguns anos, a enfermagem brasileira está em busca de reconhecimentos, direitos e condições de trabalho decentes; contudo, tem enfrentado diversas dificuldades e entraves^22^. Neste contexto, sabe-se que a valorização e o piso salarial da enfermagem envolvem aspectos históricos e morais, bem como a promoção de um trabalho digno e decente para essa classe profissional^23^. 

Riscos elevados para a instabilidade no trabalho por sintomas musculoesqueléticos foram identificados em outros estudos desenvolvidos com profissionais de enfermagem^19^ ^),(^ ^21^ e isso pode ocorrer devido aos tipos de atividades laborais realizadas, organização do trabalho e à presença dos riscos ocupacionais. 

Em uma revisão de literatura, mostrou-se que os distúrbios osteomusculares em enfermeiros associaram-se à carga física, condições organizacionais e psicossociais relacionadas ao contexto socioeconômico aumentando a vulnerabilidade ao adoecimento^24^ ^)-(^ ^25^. 

Com as análises, foi identificado que diferenças estatísticas ocorreram entre os sintomas de dor, sensação de peso e fadiga associados à alta instabilidade no trabalho. Em relação à dor, os enfermeiros(as) apresentaram um número médio de 3,72 regiões com dor na alta instabilidade no trabalho. Sobre a sensação de peso, constatou-se que os participantes tiveram um número médio de 2,60 regiões com sensação de peso na alta instabilidade no trabalho. Por fim, houve um número médio de 2,66 regiões com fadiga na alta instabilidade no trabalho entre os enfermeiros(as). 

Por meio desses resultados, pode-se afirmar que os sintomas musculoesqueléticos (dor, sensação de peso e fadiga) e alta instabilidade podem causar irregularidades entre a capacidade dos enfermeiros(as) e as atividades de trabalho, interferindo negativamente na prática laboral. 

Sobre a ocorrência da instabilidade no trabalho e a presença de sintomas musculoesqueléticos nos últimos 12 meses, identificou-se que a maioria dos(as) enfermeiros(as) teve um alto risco para a instabilidade, apesar de não apresentarem associação estatisticamente significativa entre as variáveis. Todavia, a presença de sintomas musculoesqueléticos pode estar relacionada ao maior risco de instabilidade no trabalho^7^ ^)-(^ ^8^ e, além disso, à importância de aprofundar em estudos relacionados ao impacto da instabilidade no trabalho e o absenteísmo.

Como limitações, o estudo do tipo transversal não estabelece relação entre causa e efeito das variáveis estudadas; a utilização de questionário de autopreenchimento pode ocasionar interferência de fatores não controlados e a inviabilidade de comparar alguns resultados com outros estudos científicos relacionados à instabilidade no trabalho e sintomas musculoesqueléticos entre enfermeiros(as) da APS. A amostragem por conveniência restringiu a generalização dos resultados, mas foi adotada pela viabilidade de acesso aos participantes e pela facilidade de coleta em ambiente de trabalho, permitindo reunir dados relevantes sobre a instabilidade no trabalho de enfermeiros da Atenção Primária à Saúde. Além disso, a autodeclaração dos sintomas nos últimos três meses pode envolver viés de memória, influenciando a precisão das informações sobre os sintomas relatados e provocando também limitação temporal. Já a restrição geográfica limita a generalização dos resultados, uma vez que as condições de trabalho e os vínculos laborais podem variar entre diferentes regiões do país. 

Contudo, este estudo trouxe avanços importantes ao demonstrar a complexa relação entre a instabilidade no trabalho e os distúrbios osteomusculares em enfermeiros(as) da APS no Estado de São Paulo. A pesquisa aprofundou a compreensão dos fatores individuais e laborais que influenciam a prevalência e gravidade dos sintomas musculoesqueléticos, como dor, formigamento e sensação de peso, evidenciando, por exemplo, a influência da idade, do IMC, da renda familiar e da carga horária. Além disso, o estudo destacou que a instabilidade no trabalho está associada aos diferentes perfis de sintomas e que a identificação pode ser uma ferramenta estratégica para prevenir o absenteísmo, o abandono profissional e a aposentadoria precoce, oferecendo subsídios para a criação de políticas e intervenções que promovam melhores condições laborais e a saúde dos profissionais de enfermagem.

## Conclusão

O estudo analisou a ocorrência da instabilidade no trabalho em enfermeiros(as) da APS do Estado de São Paulo e a associação com distúrbios osteomusculares e seu impacto no absenteísmo. 

Dentre os sintomas musculoesqueléticos entre os enfermeiros(as) com instabilidade no trabalho, a pesquisa mostrou que a dor no pescoço, formigamento e dormência nos punhos/mãos, sensação de peso e fadiga nos ombros ocorreram em maior prevalência.

As análises estatísticas dos enfermeiros(as) com instabilidade no trabalho mostraram que ocorreu associação entre renda familiar e instabilidade no trabalho. 

Em relação aos sintomas musculoesqueléticos (dor, sensação de peso e fadiga), ocorreram diferenças na alta instabilidade no trabalho. 

Por meio deste estudo, conclui-se que os sintomas musculoesqueléticos e a instabilidade no trabalho correlacionaram-se com as condições e os comportamentos individuais e as condições de trabalho dos(as) enfermeiros(as) da APS. Além disso, identificar previamente a instabilidade no trabalho entre essa categoria profissional pode minimizar a redução da capacidade do trabalho e, consequentemente, evitar o absenteísmo, o abandono da profissão e a aposentadoria precoce pela ocorrência dos sintomas musculoesqueléticos. Destaca-se que identificar a instabilidade no trabalho é fundamental para orientar ações e estratégias voltadas à melhoria das condições laborais dos profissionais de enfermagem, além de subsidiar políticas de prevenção e promoção da saúde ocupacional voltadas aos enfermeiros.

## Data Availability

Todos os dados gerados ou analisados durante este estudo estão incluídos neste artigo publicado.

# Translation: Inestabilidad laboral, trastornos musculoesqueléticos y absentismo en enfermeras de atención primaria*

**DOI:** 10.1590/1518-8345.8307.4938

**Authors:** Heloisa Ehmke Cardoso dos Santos, Rafael de Souza Petersen, Fernanda Ludmilla Rossi Rocha, Lacir José Santin, Maria Helena Palucci Marziale

**Affiliations:** 1 Universidade de São Paulo, Escola de Enfermagem de Ribeirão Preto, Centro Colaborador de la OPS/OMS para el Desarrollo de la Investigación en Enfermería, Ribeirão Preto, SP, Brasil.; 2 Fundação Oswaldo Cruz, Brasília, DF, Brasil

**Keywords:** Seguridad del Empleo, Trastornos de Traumas Acumulados, Atención Primaria de Salud, Enfermería, Salud Laboral, Absentismo

## Abstract

**Objetivo:**

analizar la asociación entre la inestabilidad laboral, los trastornos musculoesqueléticos y el absentismo en enfermeros de la Atención Primaria de Salud.

**Método:**

estudio metodológico, transversal, cuantitativo, realizado con 286 enfermeros y enfermeras de la Atención Primaria de Salud. Se utilizaron el Cuestionario Sociodemográfico y Ocupacional y la versión brasileña de la *Nurse-Work Instability Scale*. La recopilación de datos se realizó a través de plataformas digitales, mediante estadística descriptiva, pruebas estadísticas y modelos de regresión (p<0.05). Para la elaboración del estudio, se utilizó el programa *Strengthening the Reporting of Observational studies in Epidemiology* (STROBE).

**Resultados:**

participaron 286 enfermeros en el estudio, pero 143 (50,0%) presentaron inestabilidad en el trabajo, de los cuales el 9,8% eran de bajo riesgo, el 32,9% de riesgo medio y el 57,3% de alto riesgo. Existía una asociación significativa entre alta inestabilidad y síntomas musculoesqueléticos como dolor, sensación de pesadez y fatiga, que afectaban a múltiples regiones del cuerpo. Se observó una mayor inestabilidad entre los profesionales con mayores ingresos familiares. Los trastornos musculoesqueléticos fueron la principal causa del absentismo, aunque no existía una asociación estadística entre la inestabilidad y las bajas laborales por enfermedad.

**Conclusión:**

la inestabilidad laboral entre enfermeros y enfermeras de la Atención Primaria de Salud fue prevalente y está relacionada con factores ocupacionales y físicos, lo que requiere acciones para preservar la capacidad laboral.

## Destacados

**(1)** Este estudio analizó la inestabilidad laboral y dolores musculoesqueléticas en la Atención Primaria de Salud.

**(2)** La Inestabilidad se asoció con dolor dolor de cuello y un mayor ausentismo entre enfermeros.

**(3)** Una alta inestabilidad se asoció con dolor, pesadez y fatiga en varias regiones del cuerpo.

**(4)** Los síntomas musculoesqueléticos fueron las principales causas de baja por enfermedad.

**(5)** Los resultados impactan en las políticas sanitarias y la gestión del trabajo en enfermería.

## Introducción

El trabajo humano es uno de los principales motores del desarrollo social y económico, proporcionando al individuo no solo sustento, sino también importantes relaciones sociales y oportunidades de crecimiento profesional. En este contexto, la salud y la seguridad en el trabajo, así como la capacidad funcional de los trabajadores, son determinantes para la sostenibilidad de los sistemas productivos y para el bienestar social^1^.

La enfermería, como profesión central en el área de la salud, desempeña un papel fundamental en la promoción de la calidad de vida y la atención integral de las personas, trabajando en diferentes niveles del sistema de salud, especialmente en la Atención Primaria de Salud (APS)^2^.

En Brasil, los profesionales de enfermería representan la segunda mayor categoría de trabajadores de nivel en salud, siendo esenciales para la operativización de las políticas públicas y para garantizar el acceso universal e integral a los servicios de salud^3^. Sin embargo, estos profesionales a menudo se enfrentan a una sobrecarga de trabajo, múltiples funciones y demandas crecientes, factores que incrementan el riesgo de enfermedades físicas y mentales, especialmente en la APS, donde las condiciones laborales y los desafíos organizativos afectan directamente a su salud^4^. 

Además, un estudio internacional demostró que factores como las largas jornadas laborales, una mayor carga física y una menor autonomía profesional pueden causar una mayor vulnerabilidad a la aparición de trastornos musculoesqueléticos, evidenciando la influencia del contexto socioeconómico en la aparición de estos trastornos^5^.

Los riesgos laborales en enfermería van desde factores físicos, químicos y biológicos hasta riesgos ergonómicos y psicosociales. Los trastornos musculoesqueléticos relacionados con el trabajo (DORT) son especialmente destacados, causados por posturas inadecuadas, esfuerzos repetitivos y largas jornadas laborales, que conllevan limitaciones funcionales e impacto en la capacidad laboral^6^. Al mismo tiempo, los riesgos psicosociales, como el estrés laboral, el acoso, la inseguridad y la falta de apoyo, han sido reconocidos como factores importantes de la enfermedad mental entre las enfermeras, contribuyendo al agotamiento, la ansiedad y la depresión^7^.

En el entorno laboral, el periodo previo a la discapacidad laboral puede ocurrir cuando el trabajador empieza a presentar dificultades para realizar actividades laborales, y en este momento son necesarias intervenciones para prevenir la baja e incluso la pérdida de empleo^8^ ^)-(^ ^9^. Así, la inestabilidad laboral puede verse como un estado transitorio y potencialmente reversible si se identifica y trata a tiempo, promoviendo beneficios en los ámbitos psicosocial y económico para el trabajador y la institución empleadora^9^, minimizando las bajas y reduciendo la capacidad laboral^10^.

Dada la relevancia de la enfermería en la APS y los impactos negativos de las condiciones laborales en la salud y productividad de estos profesionales, es fundamental investigar la inestabilidad laboral, especialmente en lo que respecta a trastornos musculoesqueléticos y absentismo. Aunque existen estudios sobre riesgos laborales y trastornos musculoesqueléticos en enfermeros, la inestabilidad laboral sigue siendo un tema poco explorado, especialmente en la APS. Esta brecha de conocimiento limita la comprensión de cómo las relaciones laborales precarias y las condiciones laborales inestables pueden afectar la salud física y mental de estos profesionales, afectando su capacidad para trabajar y el absentismo.

Por ello, este estudio tuvo como objetivo analizar la asociación entre la inestabilidad laboral, los trastornos musculoesqueléticos y el absentismo en enfermeros de la Atención Primaria de Salud.

## Método

### Diseño del estudio

Este es un estudio realizado en dos etapas procedimentales: la primera consistió en un estudio metodológico para la construcción y validación de un cuestionario y la segunda en un estudio observacional, descriptivo y transversal, con un enfoque cuantitativo de los datos, y para la elaboración del estudio se utilizó el *Strengthening the Reporting of Observational Studies in Epidemiology* (STROBE). 

La primera etapa correspondió a la elaboración y validación de un cuestionario sociodemográfico y ocupacional para enfermeros y enfermeras de la Atención Primaria de Salud, basado en las referencias de Polit y Beck^11^ y Pasquali^12^. Inicialmente, la versión preliminar del instrumento se elaboró basándose en una revisión bibliográfica y principios teóricos de formulación, estructura y semántica de ítems. A continuación, el instrumento fue sometido a validación de contenidos por un comité de siete expertos con experiencia en salud laboral, trastornos musculoesqueléticos y salud mental. La validación se realizó en tres rondas, con análisis de la relevancia de los ítems y cálculo del Índice de Validez de Contenido (IVC), obteniendo un IVC final de 1,0. Posteriormente, se realizó una prueba previa con 30 enfermeros (as) de la APS para verificar la claridad, aplicabilidad y equivalencia semántica y cultural del cuestionario sociodemográfico y ocupacional. Los participantes del pretest fueron incluidos en la muestra final del estudio y los resultados confirmaron la adecuación del cuestionario para su aplicación a gran escala. La segunda fase consistió en un estudio observacional, descriptivo y transversal, con un enfoque cuantitativo de los datos, en el que se utilizaron los siguientes cuestionarios para la recogida: cuestionario sociodemográfico y ocupacional (Quest APS - sociodemográfico ocupacional) y Escala de Inestabilidad Laboral en Enfermería (EIT-enfermería).

### Lugar del estudio

Unidades de Atención Primaria de Salud en el estado de São Paulo, Brasil, desde el 27 de julio de 2023 hasta el 30 de septiembre de 2023.

### Población y muestra

La población estaba compuesta por enfermeros y enfermeras que trabajaban en unidades de Atención Primaria de Salud en el Estado de São Paulo. La muestra del estudio para la selección de participantes fue obtenida por conveniencia^11^ cumpliendo los siguientes criterios de inclusión establecidos para el estudio: enfermeros y enfermeras, sin límite de edad, que trabajaran en atención directa o en la gestión de Unidades Básicas de Salud (UBS) o Unidades de Salud Familiar (USF) en el Estado de São Paulo. Se incluyeron enfermeras que habían trabajado en enfermería durante más de un año y no tenían otra ocupación fuera del área, que habían tenido un trastorno musculoesquelético en los últimos tres meses y con al menos un episodio de dos horas o más^8^. Las enfermeras que realizaron otra ocupación fuera del área de enfermería fueron excluidas de la muestra.

Cabe destacar que no se realizó ningún cálculo previo del tamaño de la muestra, ya que se trataba de muestreo por conveniencia, basado en la disponibilidad y adhesión de los participantes durante el periodo de recogida de datos.

Las invitaciones para participar en el estudio fueron enviadas por correo electrónico por el Consejo Regional de Enfermería de São Paulo (COREN/SP).

### Variables del estudio

Las variables de resultado del estudio evaluadas corresponden al número total de sitios en el cuerpo en los que los(as) enfermeros(as) manifiestaron el síntoma referido. 

Los síntomas evaluados fueron dolor, entumecimiento, fatiga, hormigueo, sensación de pesadez y otros. La clasificación final de la Escala de Inestabilidad Laboral (EIT-Enfermería) también se evaluó como resultado, con respuestas bajas/medias y altas. Las siguientes se evaluaron como variables independientes del estudio para todos los resultados: edad (en años); sexo (masculino, femenino); ingresos familiares (hasta 3 salarios mínimos; superiores a 3 a 7 salarios mínimos; superiores a 7 salarios mínimos); índice de masa corporal (IMC) (kg/m²); ejercicio físico (sí, no); trabaja como enfermero clínico/gerente técnico (sí, no); trabaja como responsable/supervisor de atención primaria (sí, no); tipo de contrato (Consolidación de las Leyes del Trabajo - CLT, otro); trabaja en Unidad Básica de Salud/Unidad de Salud de la Familia - UBS/USF (sí, no); duración laboral en atención primaria de salud (años); tiempo en la profesión de enfermería (años); carga semanal de trabajo (hasta 36 horas, 40 horas, más de 40 horas); trabaja en el turno de mañana/tarde (sí, no), ausencia laboral por síntomas musculoesqueléticos (sí, no); ausencia laboral por ansiedad (sí, no); ausencia laboral por depresión (sí, no).

### Recogida de datos

La recogida de datos se realizó de forma remota a través de la plataforma *Research Electronic Data Capture* (REDCap)^13^, durante los meses de julio a septiembre de 2023. Enfermeros y enfermeras con registro activo y correos electrónicos válidos en el COREN/SP fueron invitados a participar en el estudio mediante una única invitación: por correo electrónico institucional. El cuestionario permaneció disponible para respuesta entre el 27 de julio y el 30 de septiembre de 2023.

Se utilizaron dos instrumentos: (1) un cuestionario sociodemográfico y ocupacional, elaborado y compuesto por 22 ítems, nueve de los cuales estaban relacionados con características demográficas y trece con aspectos ocupacionales, absentismo y presencia de síntomas musculoesqueléticos y (2) la versión nacional de la *Nurse-Work Instability Scale* (Nurse-WIS), traducida y validada en Brasil^14^. La escala, compuesta por 20 afirmaciones verdadero/falsas, identifica el nivel de inestabilidad laboral entre profesionales con trastornos musculoesqueléticos, clasificando el riesgo como bajo (<7 puntos), medio (7-13) o alto (≥14 puntos). La versión brasileña tenía un índice de fiabilidad de 0,931. El uso de la escala Nurse-WIS fue previamente autorizado por el grupo de derechos de autor, vinculado a la Universidad de Leeds, Reino Unido.

### Análisis de datos

El análisis estadístico utilizó modelos de la clase GAMLSS (*Generalized Additive Models for Location, Scale and Shape*), que permiten modelar distribuciones paramétricas de la variable de respuesta con variables independientes de forma paramétrica o no paramétrica^15^. Este enfoque abarca diferentes tipos de distribuciones (continuas, discretas, asimétricas o con colas pesadas), considerando hasta cuatro parámetros: posición (μ), escala (σ), asimetría (υ) y kurtosis (τ). Cada parámetro está asociado a un conjunto de variables independientes, a través de funciones de enlace específicas. Este enfoque ofrece un análisis más exhaustivo que se ajusta a la naturaleza de los datos recogidos.

La selección de la distribución de la variable respuesta y las variables independientes estuvo guiada por el Criterio de Información de Akaike (AIC)^16^, con una penalización estándar (k=2) para la elección de resultados y k=4 para la selección de predictores, como sugiere la literatura. También se consideró el criterio BIC (k=log(n))^17^.

La adecuación de los modelos se verificó mediante la prueba de normalidad de Shapiro-Wilk aplicada a los residuos. Se estimaron medidas de asociación como el aumento relativo (AR = (exp(β) - 1) × 100) y la razón de probabilidades. Las correlaciones entre variables numéricas y resultados se analizaron utilizando el coeficiente de Spearman y la comparación entre variables nominales y resultados se realizó mediante la prueba de Kruskal-Wallis, la prueba de chi-cuadrado de Pearson y la prueba exacta de Fisher^18^.

Los análisis se realizaron utilizando el *software* R (versión 4.3.1), adoptando un nivel de significación del 5% (α = 0,05). Los resultados se presentaron en tablas.

### Consideraciones éticas

El estudio se llevó a cabo conforme a las directrices éticas nacionales e internacionales y fue aprobado por el Comité de Ética en la Investigación de la Facultad de Enfermería de la Universidad de São Paulo en Ribeirão Preto bajo el protocolo CAAE: 57568822.0.0000.5393. Se siguió la Resolución 466/12 relativa a los estándares éticos para la investigación que involucra a seres humanos^19^. Todos los participantes que consintieron participar en el estudio firmaron el Formulario de Consentimiento Informado (FCI) y se garantizó su anonimización y confidencialidad de los datos.

## Resultados

Un total de 286 enfermeros y enfermeras participaron en el estudio, sin embargo, 143 (50,0%) presentaron inestabilidad laboral, 14 (9,80%) de bajo riesgo, 47 (32,90%) de riesgo medio y 82 (57,30%) de alto riesgo.

Entre los enfermeros y enfermeras, 125 (87,40%) eran mujeres, 77 (54,60%) estaban casadas o vivían con pareja, 99 (69,70%) eran blancas, 68 (47,90%) no tenían hijos, 62 (43,70%) tenían ingresos entre cuatro y seis salarios mínimos y 84 (58,70%) no ejercían regularmente. La edad media de los trabajadores era de 38,90 años y el IMC medio era de 28,22 kg/m².

En cuanto a los datos ocupacionales, 124 (86,70%) participantes trabajaron como enfermeros(as) clínicos(as) y 35 (24,50%) como gestores de atención primaria, 71 (50,00%) fueron contratados bajo el régimen legal, 44 (34,60%) trabajaron en la ciudad de São Paulo, 70 (49,00%) trabajaron en UBS y 87 (60,80%) en USF; la carga de trabajo semanal más frecuente era de 40 horas por semana (104 - 73,20%) y 121 (84,60%) trabajaban por la mañana. De las enfermeras, 14 (9,80%) tenían otro contrato laboral y la mayoría (7 - 49,90%) trabajaba como enfermero(a) clínico(a).

Entre las 143 enfermeros y enfermeras que informaron de la presencia de dolor, 101 (70,60%) lo reportaron en el cuello, 89 (62,20%) en los hombros, 71 (49,70%) en la parte superior de la espalda, 11 (7,70%) en los codos, 51 (35,70%) en las muñecas/manos, 58 (40,60%) en la zona lumbar, 27 (18,90%) en cadera/muslos, 43 (30,10%) en las rodillas, 32 (22,40%) en tobillos/pies, nueve (6,30%) en otra región.

En cuanto al hormigueo, 15 (10,50%) profesionales lo reportaron en el cuello, 13 (9,10%) en los hombros, cuatro (2,80%) en la parte superior de la espalda y los codos, respectivamente, 59 (41,30%) en las muñecas/manos, nueve (6,30%) en la parte superior de la espalda, siete (4,90%) en cadera/muslos, cinco (3,50%) en las rodillas, 31 (21,70%) en los tobillos/pies y ocho (5,6%) en otra región.

De los enfermeros y enfermeras que manifestaron entumecimiento en las regiones anatómicas, 11 (7,70%) lo presentaron en el cuello, ocho (5,60%) en los hombros, cuatro (2,80%) en la parte superior e baja de la espalda y rodillas, respectivamente, cinco (3,50%) en los codos, 45 (31,50%) en las muñecas/manos, seis (4,20%) en cadera/muslos y en otra región, respectivamente y 24 (16,80%) en los tobillos/pies.

En cuanto a la sensación de pesadez en las regiones anatómicas, 64 (44,80%) de enfermeros(as) informaron de este síntoma en el cuello, 88 (61,50%) en los hombros, 49 (34,30%) en la parte superior de la espalda, tres (2,10%) en los codos, diez (7,00%) en las muñecas/manos, 31 (21,70%) en la zona lumbar, 24 (16,80%) en cadera/muslos, 17 (11,90%) en las rodillas, 34 (23,80%) en los tobillos/pies y cinco (3,50%) en otra zona del cuerpo.

En cuanto a la presencia de fatiga, 57 (39,90%) enfermeros(as) la reportaron en el cuello, 68 (47,60%) en los hombros, 53 (37,10%) en la parte superior de la espalda, seis (4,20%) en los codos, 15 (10,50%) en las muñecas/manos, 38 (26,60%) en la zona lumbar, 24 (16,80%) en cadera/muslos, 21 (14,70%) en las rodillas, 31 (21,70%) en los tobillos/pies y 14 (9,80%) en otra región.

De los participantes que informaron de la presencia de otro síntoma, cinco (3,50%) lo reportaron en el cuello y la parte baja de la espalda, respectivamente; siete (4,90%) en los hombros y la parte superior de la espalda, respectivamente; dos (1,40%) en los codos; uno (0,70%) en las muñecas/manos, cuatro (2,8%) en cadera/muslos y tobillos/pies, respectivamente; seis (4,20%) en las rodillas y doce (8,4%) en otra región del cuerpo. Los síntomas reportados, en general, fueron: dolor de cabeza, convulsiones, dispnea, insomnio, taquicardia, mareos, tortícolis, vértigo, dolor precordial (en el pecho).

Los datos de la Tabla 1 muestran los valores de las pruebas bivariantes de las características sociodemográficas y ocupacionales y de la inestabilidad laboral de enfermeros(as). Sin embargo, cabe destacar que no todos los participantes respondieron a todas las preguntas del cuestionario.

**Tabla 1:** Distribución de los valores obtenidos según pruebas bivariantes de características sociodemográficas y ocupacionales y resultados de inestabilidad laboral de enfermeros y enfermeras que trabajan en la Atención Primaria de Salud paulista (n = 143). Estado de São Paulo, Brasil, 2023

	Alto	Bajo|Medio	Prueba estadística	Valor p
**Total**	82	61		
**Sexo**			Chi-Cuadrado (1 df*) = 0,12	0,730
Femenino	71 (56,8%)	54 (43,2%)		
Masculino	11 (61,1%)	7 (38,9%)		
**Ingresos familiares** ^†^ (salario mínimo)			Chi-Cuadrado (1 df*) = 11,81	0,003
Entre 1 y 3	12 (57,1%)	9 (42,9%)		
Entre 4 y 6	26 (41,9%)	36 (58,1%)		
Acima de 7	43 (72,9%)	16 (27,1%)		
**Práctica de ejercicio físico**			Chi-Cuadrado (1 df*) = 0,4	0,529
No	50 (59,5%)	34 (40,5%)		
Sí	32 (54,2%)	27 (45,8%)		
**Enfermero(a) asistencial**			Chi-Cuadrado (1 df*) = 0,2	0,658
No	10 (62,5%)	6 (37,5%)		
Si	72 (56,7%)	55 (43,3%)		
**Gestor de atención primaria**			Chi-Cuadrado (1 df*) = 0,47	0,493
No	62 (59%)	43 (41%)		
Sí	20 (52,6%)	18 (47,4%)		
**Tipo de contrato**			Exacto de Fisher	0,075
CLT^‡^	44 (67,7%)	21 (32,3%)		
Estatutario	35 (49,3%)	36 (50,7%)		
Otros	3 (50%)	3 (50%)		
UBS^§^			Chi-Cuadrado (1 df*) = 3,2	0,074
No	36 (50%)	36 (50%)		
Sí	46 (64,8%)	25 (35,2%)		
USF^||^			Chi-Cuadrado (1 df*) = 1	0,317
No	35 (62,5%	21 (37,5%)		
Sí	47 (54%)	40 (46%)		
**Carga semanal de trabajo (horas)**			Chi-Cuadrado (2 df*) = 0,36	0,835
Hasta 36	17 (60,7%)	11 (39,3%)		
40	59 (56,7%)	45 (43,3%)		
Más de 40	5 (50%)	5 (50%)		
**Turno de trabajo - mañana**			Chi-Cuadrado (1 df*) = 0,08	0,773
No	12 (54,5%)	10 (45,5%)		
Sí	70 (57,9%)	51 (42,1%)		
**Turno de trabajo - tarde**			Chi-Cuadrado (1 df*) = 0,16	0,690
No	14 (53,8%)	12 (46,2%)		
Sí	68 (58,1%)	49 (41,9%)		
**Ansiedad**			Chi-Cuadrado (1 df*) = 0,47	0,495
No	74 (56,5%)	57 (43,5%)		
Sí	8 (66,7%)	4 (33,3%)		
**Depresión**			Chi-Cuadrado (1 df*) = 0,01	0,942
No	75 (57,3%)	56 (42,7%)		
Sí	7 (58,3%)	5 (41,7%)		

*Df = *Degrees of freedom* (grados de libertad); ^†^Ingreso familiar = Salario mínimo actual de R$ 1.320,00, Brasil, 2023; ^‡^CLT = Consolidación de las Leys del Trabajo; ^§^UBS = Unidade Básica de Salud; ^||^USF = Unidade de Salud Familiar

Para el análisis de los datos de clasificación EIT, se realizó una agrupación entre las categorías baja y media, debido a un pequeño número de encuestados en la categoría de baja inestabilidad laboral. Entre los enfermeros y enfermeras que participaron en el estudio, la mayoría mostró alta inestabilidad en el trabajo. 

En cuanto a las variables analizadas, hubo una asociación entre ingresos familiares e inestabilidad laboral (p=0,003) y, entre los participantes, la mayoría presentaba alta inestabilidad laboral y ingresos familiares superiores a 7 salarios mínimos (43-72,9%), lo que mostró que cuanto mayor era el ingreso, mayor era la inestabilidad laboral.

Los datos sobre síntomas musculoesqueléticos, edad, IMC, tiempo de trabajo en atención primaria y como enfermero(a) clínico(a) se presentan en la Tabla 2.

**Tabla 2:** Distribución de valores obtenidos según síntomas musculoesqueléticos, edad (en años), índice de masa corporal (kg/m²), tiempo de trabajo en atención primaria y como enfermero y enfermera clínica (en años) e inestabilidad laboral de enfermeros y enfermeras que trabajan en la Atención Primaria de Salud paulista (n = 143). Estado de São Paulo, Brasil, 2023

Variables	Clasificación EIT*	n	Media	Desviación estándar	Mínimo	Q^†^0.25	Q^†^0.5	Q^†^0.75	Máximo	Valor p
Dolor	Alto	82	3,72	1,57	1,00	3,00	3,00	5,00	8,00	0,0164
Dolor	Bajo|Medio	61	3,07	1,72	0,00	2,00	3,00	4,00	9,00	
Parestesia	Alto	82	0,95	1,15	0,00	0,00	1,00	1,75	7,00	0,0698
Parestesia	Bajo|Medio	61	0,64	0,91	0,00	0,00	0,00	1,00	5,00	
Hormigueo	Alto	82	1,22	1,23	0,00	0,00	1,00	2,00	7,00	0,0639
Hormigueo	Bajo|Medio	61	0,90	1,03	0,00	0,00	1,00	1,00	5,00	
Sensación de pesadez	Alto	82	2,60	1,68	0,00	2,00	2,00	3,00	9,00	0,0023
Sensación de pesadez	Bajo|Medio	61	1,84	1,50	0,00	1,00	1,00	3,00	8,00	
Fatiga	Alto	82	2,66	2,41	0,00	1,00	2,00	4,00	10,00	0,0233
Fatiga	Bajo|Medio	61	1,79	1,92	0,00	0,00	1,00	3,00	9,00	
Otros síntomas	Alto	82	0,43	0,86	0,00	0,00	0,00	1,00	5,00	0,2823
Otros síntomas	Bajo|Medio	61	0,30	0,67	0,00	0,00	0,00	0,00	3,00	
Edad	Alto	61	38,59	8,52	23,00	33,00	38,00	44,00	66,00	0,7547
Edad	Bajo|Medio	80	39,14	7,13	26,00	34,00	38,50	43,00	60,00	
IMC^‡^	Alto	61	27,77	5,93	18,65	22,89	27,10	31,62	42,15	0,4439
IMC^‡^	Bajo|Medio	80	28,56	5,76	19,31	24,92	27,09	30,93	48,88	
Tiempo de trabajo en la atención primaria de salud	Alto	61	9,23	7,00	0,00	3,00	9,00	13,00	40,00	0,9299
Tiempo de trabajo en la atención primaria de salud	Bajo|Medio	82	9,20	6,32	1,00	4,25	8,00	12,75	33,00	
Tiempo de trabajo como enfermero(a) asistencial	Alto	61	11,41	6,60	0,00	7,00	12,00	17,00	24,00	0,3838
Tiempo de trabajo como enfermero(a) asistencial	Bajo|Medio	82	12,73	6,55	2,00	9,00	11,50	17,00	34,00	

*EIT = Escala de Inestabilidad Laboral; ^†^Q = Cuartil; ^‡^IMC = Índice de masa corporal

Mediante pruebas estadísticas, se identificó que existían diferencias entre el dolor y la alta inestabilidad en el trabajo (p=0,0164), sensación de pesadez y alta inestabilidad en el trabajo (p=0,0023) y fatiga y alta inestabilidad en el trabajo (p=0,0233).

En cuanto al dolor, enfermeros(as) tuvieron un número medio de 3,72 regiones con dolor de alta inestabilidad en el trabajo.

En cuanto a la sensación de pesadez, se encontró que los participantes tenían un número medio de 2,60 regiones con una sensación de peso en alta inestabilidad en el trabajo.

Finalmente, también hubo un número medio de 2,66 regiones con fatiga y alta inestabilidad laboral entre los participantes. 

La Tabla 3 presenta los datos del análisis de regresión de la inestabilidad laboral en relación con el dolor, la parestesia, el hormigueo, la sensación de pesadez, fatiga, otros síntomas y los ingresos familiares.

**Tabla 3:** Análisis de regresión de la inestabilidad laboral entre enfermeros(as) que trabajan en la Atención Primaria de Salud paulista y que presentaron inestabilidad laboral (n = 143). Estado de São Paulo, Brasil, 2023

µ*	Estimación	Desviación estándar	Valor p	Pr(>|t|)^†^	Aumento relativo	Intervalo de Confianza	
(Intercepto)	-0,1169	0,5043	-0,2318	0,8171	OR^‡^	LI_OR^§^	LS_OR^||^
Dolor	0,0956	0,1564	0,6110	0,5423	1,1003	0,8098	1,4950
Parestesia	0,0506	0,2646	0,1912	0,8457	1,0519	0,6263	1,7667
Hormigueo	0,1281	0,2382	0,5378	0,5916	1,1367	0,7127	1,8129
Sensación de pesadez	0,2792	0,1966	1,4204	0,1579	1,3221	0,8994	1,9436
Fatiga	-0,0160	0,1280	-0,1250	0,9007	0,9841	0,7657	1,2648
Otros síntomas	0,3329	0,2595	1,2832	0,2018	1,3951	0,8390	2,3198
Ingreso familiar^¶^ entre 1 y 3 salarios mínimos	-1,2242	0,6027	-2,0310	0,0443	0,2940	0,0902	0,9581
Ingreso familiar^¶^ entre 4 y 6 salarios mínimos	-1,4687	0,4216	-3,4832	0,0007	0,2302	0,1008	0,5261

*µ = Letra griega “mi” (media de población); ^†^Pr(>|t|) = Valor p; ^‡^OR = *Odds Ratio* (razón de probabilidades); ^§^LI_OR = Límite inferior del intervalo de confianza del *odds ratio*; ^||^LS_OR = Límite superior del intervalo de confianza del *odds ratio*; ^¶^Ingreso familiar = Salario mínimo actual de R$ 1.320,00, Brasil, 2023

A partir del análisis de regresión realizado, se identificó que los participantes con ingresos familiares entre uno y tres salarios mínimos tenían una puntuación media 70,60% (1 - 0,2940) (p=0,0443) menor en inestabilidad laboral que aquellos con ingresos superiores a siete salarios mínimos. Además, los(as) enfermeros(as) con ingresos familiares entre cuatro y seis salarios mínimos mostraron una puntuación media del 76,98% (1 - 0,2302) (p=0,0007) menor en inestabilidad que aquellas con ingresos superiores a siete salarios mínimos.

La siguiente Tabla 4 muestra el absentismo entre los(as) enfermeros(as) en los últimos 12 meses, manifestado a través de síntomas como la inestabilidad laboral.

**Tabla 4:** Distribución de las pruebas obtenidas según la presencia de síntomas musculoesqueléticos e inestabilidad laboral de enfermeros y enfermeras que trabajan en Atención Primaria de Salud paulista (n = 143). Estado de São Paulo, Brasil, 2023

	Alto	Bajo| Medio	Prueba estadística	Valor p
**Síntomas musculoesqueléticos en los últimos 12 meses**			Chi-Cuadrado (1 df*) = 1,43	0,231
Sí	29 (65,9%)	15 (34,1%)		
No	53 (53,5%)	46 (46,5%)		

*Df = *Degrees of freedom* (grados de libertad)

En cuanto a la presencia de síntomas musculoesqueléticos en los últimos 12 meses e inestabilidad laboral, se encontró que no hubo significación mediante la prueba estadística; Sin embargo, de los 29 (65,9%) enfermeros y enfermeras ausentes, la mayoría tenía un alto riesgo de inestabilidad laboral.

## Discusión

A través del estudio, se descubrió que la mayoría de los participantes eran mujeres, casadas, blancas, sin hijos, con una edad media de 38,90 años y un ingreso mensual entre 4 y 6 salarios mínimos. En un estudio realizado entre enfermeros(as) con inestabilidad laboral, se evidenció que la población estudiada era mayoritariamente femenina, lo que confirma la predominancia de mujeres en el trabajo de enfermería. En cuanto al peso y al IMC, la media fue de 77,38 kg y 28,22 kg/m², respectivamente^20^; además, la mayoría de los participantes no practicaban ejercicio físico de forma regular, siendo clasificados como con sobrepeso^3^.

Datos similares sobre sexo y edad se encontraron en un estudio que evaluó la inestabilidad laboral entre enfermeros(as) en un hospital universitario brasileño^21^.

En el ámbito del trabajo de la enfermería, la mayoría de los(as) enfermeros(as) trabajaban en cuidados de la APS, en la ciudad de São Paulo y bajo el régimen estatutario legal. En cuanto al lugar de trabajo, la mayoría de enfermeros y enfermeras en São Paulo trabajaban en la USF por la mañana, y la carga semanal más frecuente era de 40 horas semanales. Siguiendo con las características ocupacionales, la minoría tenía otra relación laboral. También se encontró que enfermeros y enfermeras tenían una media de 9,21 años de trabajo en la APS y 12,17 años trabajando como enfermeros(as).

Entre los síntomas musculoesqueléticos de enfermeros(as) con inestabilidad en el trabajo, dolor de cuello, hormigueo y entumecimiento en las muñecas/manos, sensación de pesadez y fatiga en los hombros fueron los más reportados. Los otros síntomas reportados fueron dolor corporal, irritabilidad, ardor corporal, dolor de cabeza, taquicardia, vértigo, tortícolis, ansiedad, convulsiones, disnea, fatiga, insomnio, lesión por herpes zóster y dolor lumbar.

No se encontraron estudios sobre la inestabilidad laboral entre enfermeros(as) de la APS; sin embargo, en un estudio realizado con profesionales de enfermería de un hospital universitario en Brasil, se encontró que la región lumbar, la parte superior de la espalda, los hombros, los tobillos y los pies, cuello y rodillas eran las regiones más afectadas por trastornos musculoesqueléticos, en ese orden^21^.

En este estudio, se encontró que existía una asociación entre ingresos familiares e inestabilidad laboral y, entre las enfermeras, la mayoría tenía alta inestabilidad laboral y ingresos familiares superiores a 7 salarios mínimos. El análisis de regresión encontró que los participantes con ingresos familiares entre 1 y 3 salarios mínimos tenían una puntuación media un 70,60% menor en inestabilidad laboral que aquellos con ingresos superiores a 7 salarios mínimos y los(as) que perciben ingresos familiares de entre 4 y 6 salarios mínimos mostraron una puntuación media del 76,98% menor en inestabilidad que los(as) con ingresos superiores a 7 salarios mínimos.

Estos datos permiten afirmar que que cuanto mayor es el ingreso, mayor es la probabilidad de inestabilidad laboral entre los(as) enfermeros(as) y esta situación puede deberse al bajo salario de la enfermería y a la necesidad de tener más de un empleo para mejorar los ingresos.

Desde hace algunos años, la enfermería brasileña busca reconocimiento, derechos y condiciones laborales dignas; sin embargo, ha enfrentado varias dificultades y obstáculos^22^. En este contexto, se sabe que la valorización y el salario mínimo de la enfermería implican aspectos históricos y morales, así como la promoción de un trabajo digno y digno para esta clase profesional^23^.

Se han identificado altos riesgos de inestabilidad laboral debido a síntomas musculoesqueléticos en otros estudios realizados con profesionales de enfermería^19^ ^),(^ ^21^ y esto puede darse debido a los tipos de actividades laborales realizadas, la organización del trabajo y la presencia de riesgos laborales.

En una revisión bibliográfica, se demostró que los trastornos musculoesqueléticos en enfermeros(as) estaban asociados con la carga de trabajo física, condiciones organizativas y psicosociales relacionadas con el contexto socioeconómico, aumentando la vulnerabilidad a la enfermedad^24^ ^)-(^ ^25^.

Con los análisis se identificó que se observaron diferencias estadísticas entre los síntomas de dolor, sensación de pesadez y fatiga asociados a una alta inestabilidad en el trabajo. En cuanto al dolor, los(as) enfermeros(as) tuvieron un promedio de 3,72 regiones con dolor en alta inestabilidad laboral. En cuanto a la sensación de peso, se encontró que los participantes tenían un número medio de 2,60 regiones con una sensación de peso en alta inestabilidad en el trabajo. Finalmente, hubo un número medio de 2,66 regiones con fatiga y alta inestabilidad laboral entre enfermeros y enfermeras.

A partir de estos resultados, se puede afirmar que los síntomas musculoesqueléticos (dolor, sensación de pesadez y fatiga) y la alta inestabilidad pueden causar irregularidades entre la capacidad y las actividades laborales de los(as) enfermeros(as), interfiriendo negativamente en la práctica laboral.

En cuanto a la aparición de inestabilidad en el trabajo y la presencia de síntomas musculoesqueléticos en los últimos 12 meses, se identificó que la mayoría de los(as) enfermeros(as) tenían un alto riesgo de inestabilidad, aunque no presentaron una asociación estadísticamente significativa entre las variables. Sin embargo, la presencia de síntomas musculoesqueléticos puede estar relacionada con un mayor riesgo de inestabilidad en el trabajo^7^ ^)-(^ ^8^ y, además, con la importancia de estudios adicionales relacionados con el impacto de la inestabilidad laboral y el absentismo.

Como limitaciones, el estudio transversal no establece una relación entre causa y efecto de las variables estudiadas; el uso de un cuestionario autoadministrado puede causar interferencias de factores no controlados y la inviabilidad de comparar algunos resultados con otros estudios científicos relacionados con la inestabilidad laboral y los síntomas musculoesqueléticos entre enfermeras de la APS. El muestreo por conveniencia limitó la generalización de los resultados, pero se adoptó debido a la viabilidad de acceso a los participantes y la facilidad de recogida en el entorno laboral, lo que permitió recopilar datos relevantes sobre la inestabilidad en el trabajo de las enfermeras en Atención Primaria de Salud. Además, el autoinforme de los síntomas en los últimos tres meses puede implicar sesgo de recuerdo, influyendo en la precisión de la información sobre los síntomas reportados y también causando limitaciones temporales. La restricción geográfica, por otro lado, limita la generalización de los resultados, ya que las condiciones y las relaciones laborales pueden variar entre distintas regiones del país.

Sin embargo, este estudio aportó avances importantes al demostrar la compleja relación entre la inestabilidad laboral y los trastornos musculoesqueléticos en enfermeros(as) de la APS en el Estado de São Paulo. La investigación profundizó en la comprensión de factores individuales y laborales que influyen en la prevalencia y gravedad de los síntomas musculoesqueléticos, como dolor, hormigueo y sensación de pesadez, mostrando, por ejemplo, la influencia de la edad, el IMC, los ingresos familiares y la carga de trabajo. Además, el estudio destacó que la inestabilidad laboral está asociada a diferentes perfiles de síntomas y que la identificación puede ser una herramienta estratégica para prevenir el absentismo, el abandono profesional y la jubilación anticipada, ofreciendo subvenciones para la creación de políticas e intervenciones que promuevan mejores condiciones laborales y la salud de los profesionales de enfermería.

## Conclusión

El estudio analizó la ocurrencia de inestabilidad laboral en enfermeros(as) de PHC en el Estado de São Paulo y la asociación con trastornos musculoesqueléticos y su impacto en el absentismo.

Entre los síntomas musculoesqueléticos entre enfermeros(as) con inestabilidad laboral, la investigación mostró que el dolor de cuello, hormigueo y entumecimiento en las muñecas/manos, sensación de pesadez y fatiga en los hombros se presentaban en mayor prevalencia.

Los análisis estadísticos de enfermeros(as) con inestabilidad laboral mostraron que existía una asociación entre ingresos familiares e inestabilidad laboral.

En cuanto a los síntomas musculoesqueléticos (dolor, sensación de pesadez y fatiga), hubo diferencias en la alta inestabilidad laboral.

A través de este estudio, se concluye que los síntomas musculoesqueléticos y la inestabilidad laboral estaban correlacionados con condiciones y comportamientos individuales y con las condiciones laborales de los enfermeros y enfermeras de la APS. Además, identificar previamente la inestabilidad laboral en esta categoría profesional puede minimizar la reducción de la capacidad laboral y, en consecuencia, evitar el absentismo, el abandono de la profesión y la jubilación anticipada debido a la aparición de síntomas musculoesqueléticos. Cabe destacar que identificar la inestabilidad en el trabajo es esencial para guiar acciones y estrategias destinadas a mejorar las condiciones laborales de los profesionales de enfermería, además de subvencionar políticas de prevención y promoción de la salud laboral dirigidas a los(as) enfermeros(as).

## Data Availability

Todos los datos generados o analizados durante este estudio están incluidos en este artículo publicado.
